# Natural selection and recombination interact to structure genome‐wide variation in pines

**DOI:** 10.1111/tpj.70866

**Published:** 2026-04-14

**Authors:** Chen‐Jui Yang, Tomas Funda, Rajiv Chaudhary, Zhi‐Qiang Chen, Maximiliano Estravis Barcala, Harry Wu

**Affiliations:** ^1^ Department of Forest Genetics and Plant Physiology, Umeå Plant Science Centre Swedish University of Agricultural Sciences Linnaeus väg 6 901 83 Umeå Sweden; ^2^ Department of Forest Genetics and Regeneration Norwegian Institute of Bioeconomy Research Høgskoleveien 8 1431 Ås Norway; ^3^ State Key Laboratory of Tree Genetics and Breeding, Co‐Innovation Center for Sustainable Forestry in Southern China, College of Forestry Nanjing Forestry University Nanjing 210037 China

**Keywords:** genomic landscapes, comparative genomics, recombination, conifer genome, *Pinus*, natural selection, long gene

## Abstract

Genetic differentiation among populations often varies significantly across the genome due to factors such as selection and recombination, resulting in a heterogeneous genomic landscape. However, variation in low‐differentiation regions—genomic valleys—remains poorly understood. Moreover, most insights into plant genomic landscapes come from flowering plants, while comparable genome‐wide studies in other taxa, such as conifers, remain limited. We analyzed whole‐genome sequencing data from 100 individuals of three pine species—*Pinus banksiana*, *Pinus contorta*, and *Pinus nigra*. We found substantial genome‐wide variation in recombination rates, with intergenic regions exhibiting higher recombination than genic regions, and rates decreasing with increasing distance from genes. Recombination rate was negatively correlated with gene length, driven primarily by intron length, suggesting that long introns in conifers may promote the retention of exceptionally long genes by maintaining low recombination in these regions. Genomic scans further revealed that genomic valleys are maintained through either balancing, background, or parallel selection. Additionally, multiple forms of selection were strongly associated with local recombination rate variation, highlighting the significant role of recombination in shaping patterns of genomic differentiation. Our findings provide new insight into the evolution and maintenance of extremely long genes in conifers. Moreover, the results indicate that allopatric selection in regions of low recombination is a major force structuring genomic variation in these species.

## Significance Statement

Our findings show that pronounced recombination rate variation, especially the suppression of recombination within long intron genes, helps explain the evolution of exceptionally large and complex conifer genomes. We further demonstrate that genomic islands and valleys arise from diverse forms of selection closely tied to local recombination environments, providing new insight into how genome structure and diversity are shaped across ecologically and economically important non‐flowering plants.

## INTRODUCTION

The term ‘genomic landscape’ encompasses the complex and dynamic organization of genetic variations within an organism's genome, reflecting both the spatial arrangements and the evolutionary processes that shape it. This landscape is characterized by its heterogeneity, demonstrating the myriad factors that contribute to the differentiation and adaptation of populations over time. Various evolutionary mechanisms, including mutation, genetic drift, natural selection, gene flow, and recombination, play crucial roles in sculpting this genomic terrain, leading to distinct patterns of genetic diversity observed within and among species (Nosil et al., 2009; Burri, 2017; Ravinet et al., 2017).

Understanding these intricately intertwined processes and their roles in shaping the genomic landscape presents significant challenges. Fortunately, the recent availability of high‐resolution genomic data has enabled genome scanning and comparative genomics to emerge as powerful approaches for studying genetic divergence among populations and across species. Recent studies have identified regions of notable genetic divergence, termed ‘genomic islands of divergence’ or ‘genomic islands’, contrasted with surrounding genomic backgrounds (‘sea level’) (Cruickshank & Hahn, 2014; Han et al., 2017; Irwin et al., 2018; Sendell‐Price et al., 2020). Similarly, regions displaying low differentiation levels are referred to as ‘genomic valleys of divergence’ or ‘genomic valleys’ (Roesti et al., 2014; Sendell‐Price et al., 2020).

Genomic islands may arise from processes related to reproductive isolation and local adaptation, but they can also result from indirect selection mechanisms such as genetic hitchhiking, background selection, or intrinsic genomic features such as variation in recombination rate (Burri et al., 2015; Cruickshank & Hahn, 2014; Noor & Bennett, 2009; Nosil et al., 2009). Regardless of their origin, linkage disequilibrium (LD) plays a key role in their formation by reducing within‐population diversity and increasing between‐population differentiation. Recombination counteracts this process by breaking down LD. Therefore, characterizing genome‐wide variation in LD and recombination rates is essential for genomic scanning and comparative genomics, as these factors determine how selection on specific loci influences nearby regions, ultimately shaping genomic patterns of diversity and differentiation (Charlesworth et al., 1993; Wolf & Ellegren, 2017).

In addition to recombination rate variation *per se*, specific genomic features may actively shape local recombination landscapes. In plant genomes, transposable elements (TEs) are particularly abundant and have been shown to play a major role in structuring patterns of recombination (Bennetzen, 2000; Feschotte & Pritham, 2007; Pulido & Casacuberta, 2023; Tenaillon et al., 2010). TE insertions are often associated with epigenetic silencing mechanisms such as DNA methylation, which can promote heterochromatin formation and suppress crossover activity in surrounding regions (Baduel & Colot, 2021; Cossu et al., 2017; Kent et al., 2017). This process can generate extended regions of reduced recombination, thereby influencing patterns of genetic diversity, LD, and the efficacy of selection across the genome.

In contrast to genomic islands, which often emerge during the early stage of population divergence, genomic valleys typically become apparent only after substantial divergence has occurred. Although genomic valleys have been reported in various species (Roesti et al., 2012; Van Doren et al., 2017; Wang et al., 2016), their role in shaping the genomic landscape remains poorly understood. Potential mechanisms include parallel or balancing selection (Charlesworth, 2006; Nielsen, 2005; Roesti et al., 2012, 2014), purifying and background selection (Cvijović et al., 2018), and adaptive introgression (Christe et al., 2017; Roux et al., 2013; Zhang et al., 2023). Genomic valleys may also result from incomplete lineage sorting, where shared alleles persist during the early phase of divergence, resulting in regions with below‐background differentiation levels (Sendell‐Price et al., 2020). Furthermore, the association between genomic valleys and recombination rates variation, as well as various genomic features, remains largely unexplored, likely because previous genome scanning studies have focused on the early divergence stages associated with speciation.

In plants, genome scanning and comparative genomic studies have predominantly focused on flowering species, while research on other taxa, such as conifers, remains limited. Conifer genomes often exceed 20 Gb, which are far larger than those of most flowering plants and contain abundant and complex repetitive sequences, largely composed of TEs (Niu et al., 2022). These features have historically hindered genome assembly, producing fragmented and incomplete drafts (e.g., Wegrzyn et al., 2014; Zimin et al., 2014) and constraining comparative analyses that depend on high‐quality reference genomes. Recent advances in long‐read sequencing, chromosome conformation capture, and improved assembly algorithms now enable much more complete conifer genomes. Several conifer species, including Japanese cedar (*Cryptomeria japonica* (L.f.) D.Don; Fujino et al., 2024), Masson's pine (*Pinus massoniana* Lamb.; Chen et al., 2025), and Chinese pine (*Pinus tabuliformis* Carr.; Niu et al., 2022), have achieved chromosome‐level assemblies, greatly enhancing opportunities for comparative genomic studies.

In this study, we aim to characterize the genomic landscape, encompassing both genomic islands and genomic valleys, in conifers and to elucidate the evolutionary and genomic mechanisms underlying their formation. We analyzed whole‐genome sequences from three ecologically and economically important pine species: *Pinus banksiana* Lamb., *Pinus contorta* Dougl., and *Pinus nigra* J.F. Arnold. By integrating genome‐wide divergence analyses with recombination rate and genomic feature data, we reveal how patterns of genomic landscape relate to recombination rate variation, genomic features, and potential signatures of selection. Our findings advance the understanding of how genomic islands and valleys interact with evolutionary processes to sculpt the genomic landscape of divergence in long‐lived, non‐flowering plant lineages.

## RESULTS

We generated whole‐genome sequencing data for 100 pine trees of the three focal pine species, yielding an average of 2.16 million reads per sample (Figure S1; Table S1). The chromosome‐level assembly and annotation of *P*. *tabuliformis* was used as the reference genome (Niu et al., 2022), as it represented the most complete and well‐annotated *Pinus* genome at the onset of this study. The sister species *P*. *banksiana* and *P. contorta* belong to subgenus *Pinus*, section *Trifoliae*, with their common ancestor dating to approximately 11.2–13.4 million years ago (Jin et al., 2021). *Pinus nigra* and *P*. *tabuliformis* belong to subgenus *Pinus*, section *Pinus*, sharing a common ancestor around 21.2–25.1 million years. The divergence between sections *Pinus* and *Trifoliae* is estimated at 50.0–56.1 million years (Jin et al., 2021). The native distributions of the three focal species are shown in Figure S1.

The mean mapping rates to the reference genome were 70.7, 71.8, and 91.9, with corresponding average read mapping depths of 32.0×, 33.8×, and 44.3× for *P. banksiana*, *P. contorta*, and *P. nigra*, respectively (Table S1). A total of 1 397 232 410 SNPs were called before filtering, and 59 080 747 high‐quality SNPs were retained after applying a stringent quality control pipeline. The numbers of private and shared SNPs among the three species are shown in Figure S2. *Pinus nigra* exhibited the highest number of private SNPs, consistent with its close phylogenetic relationship with the reference genome. In contrast, the SNPs shared between *P. banksiana* and *P. contorta* were the second most abundant, reflecting their close relationship as sister species.

To assess the potential coverage bias caused by genetic distance to the reference, we compared the number of all retained sites (including SNPs and invariant sites) across genomic categories with their corresponding total lengths in the reference genome (Table S2). The results indicate that retained sites are disproportionately concentrated in genic regions, although their numbers are proportional to the reference genome's genic length distribution. This bias likely arises from sequence divergence between the focal species and the reference genome, with intergenic regions being more prone to mapping errors due to their higher sequence divergence.

### Genetic diversity and population structures

To minimize potential bias arising from genetic distance to the reference genome, we estimated nucleotide diversity (*π*) for each species using *pixy* (Korunes & Samuk, 2021), which corrects for missing data by accounting for both variant and invariant sites. Nonetheless, as noted above, estimates in intergenic regions may still be biased downward due to lower coverage. Genome‐wide *π* was highest in *P. nigra*, followed by *P. contorta* and *P. banksiana* (Table 1), likely due to more intraspecific divergence, as both species can be further classified into multiple subspecies. Furthermore, the higher estimated *π* in *P. nigra* may also be due to the phylogenetic affinity to the reference genome. Substantial heterogeneity in nucleotide diversity was observed across the genomes of all three species (Figures S3 and S4).

**Table 1:** Mean nucleotide diversity (*π*) across all sites of the three *Pinus* species

Species	*P. banksiana*	*P. contorta*	*P. nigra*
Whole genome	0.0026	0.0032	0.0037
Zero‐fold degenerate sites	0.0017	0.0019	0.0025
Four‐fold degenerate sites	0.0034	0.0039	0.0049
3′ UTRs	0.0023	0.0026	0.0033
5′ UTRs	0.0022	0.0025	0.0032
Introns	0.0022	0.0025	0.0034
Intergenic sites	0.0028	0.0034	0.0038

Among functional categories, *π* were highest at fourfold degenerate sites, followed by intergenic sites, 3′ UTRs, 5′ UTRs, and introns, and lowest at zerofold degenerate sites (Table 1). The ratio of *π* at zerofold to fourfold degenerate sites was 0.489, 0.481, and 0.502 in *P. banksiana*, *P. contorta*, and *P. nigra*, respectively. This pattern of relative levels of nucleotide diversity across site categories is broadly consistent with previous findings in pines and other eukaryotes (Andolfatto, 2005; Chen et al., 2017; Eckert et al., 2013; Larracuente et al., 2008; Wang et al., 2016).

Additionally, to compare *π* estimates derived from different analytical approaches, we reanalyzed the *P. nigra* dataset generated by Zhao et al. (2024) using the same protocols and methods applied in this study (Table S3). The results demonstrate that methodological factors, such as sequencing strategy and platform, reference genome choice, SNP filtering thresholds, and the specific statistics used for estimation, can significantly influence nucleotide diversity estimates.

**Figure 1:**
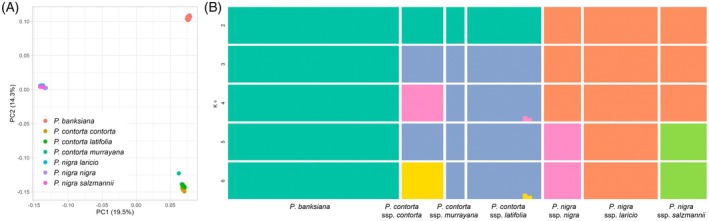
Estimates of genetic structure of the three *Pinus* species. (A) Genome‐wide PCA plot among all individuals of the three *Pinus* species. The first two principal components (PCs) are shown. Subspecies, if present, are shown in different colors. (B) Genetic structure of the three *Pinus* species estimated using ADMIXTURE under *K* = 2–6 genetic cluster models. Each vertical bar indicates a single individual, and the height of each color indicates the probability of assignment. The best‐supported model based on the cross‐validation method was *K* = 3.

Principal component analysis (PCA) revealed clear clustering patterns consistent with current species classifications, whereas subspecific differentiation was less distinct (Figure 1A). The first principal component (PC1) accounted for 19.5% of the total genetic variation, and the second (PC2) explained 14.3%. The remaining principal components each contributed approximately 5% or less. Within *P. nigra*, three distinct clusters were identified, corresponding to *P. nigra* ssp. *nigra*, ssp. *salzmannii*, and ssp. *laricio* (Figure S5B). Similarly, three clusters were detected within *P. contorta*, with *P. contorta* ssp. *contorta* clearly separated from the other two subspecies. Two individuals of *P. contorta* ssp. *murrayana*, however, grouped within the *P. contorta* ssp. *latifolia* cluster (Figure S5A).

In the clustering analysis, the optimal number of clusters (*K*) with the lowest cross‐validation error was three, while the clustering results can also be explained according to their infraspecific classification when *K* = 4, 5, and 6 (Figure 1B). Both the PCA and clustering results suggest that no recent interspecific hybrid individual was sampled in this study.

### Demographic histories

**Figure 2:**
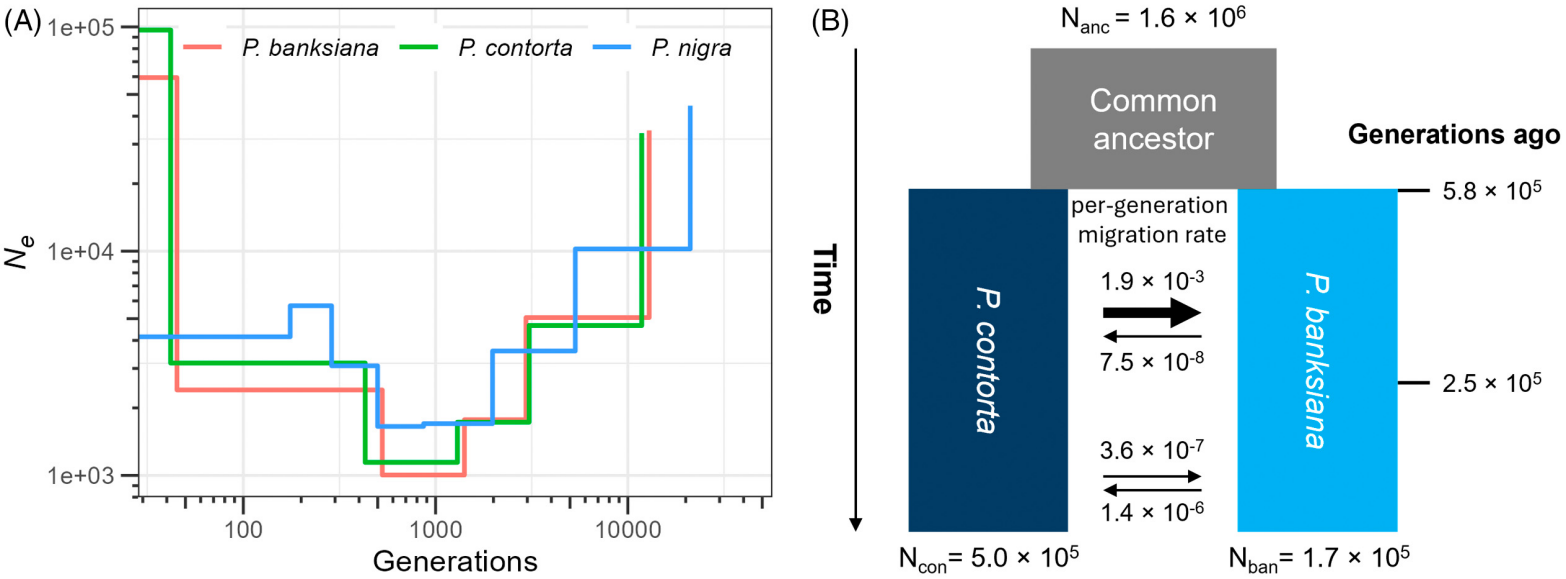
Demographic history and gene flow analyses. (A) SMC++ analysis of the dynamic changes in effective population size (*N*_e_) through time of the three *Pinus* species. (B) The best‐fitting demographic scenario of *Pinus banksiana* and *Pinus contorta* using fastsimcoal2.

To investigate the demographic histories of the three pine species, we first inferred dynamic changes in the effective population size (*N*_e_) over time for each species using Sequentially Markovian coalescent based SMC++ (Terhorst et al., 2017). The results indicated that all three pine species have undergone comparable demographic trajectories (Figure 2A). Each species experienced a consistent decline in *N*_e_, reaching its lowest level (approximately 1000–2000) around 1000–2000 generations ago, followed by an expansion approximately 400–500 generations ago. Assuming a generation time of 25 years (Brown et al., 2004), the three species experienced a bottleneck roughly 25 000–50 000 years ago, with subsequent expansion around 10 000–12 500 years ago. These findings suggest that the population sizes of all three pine species declined during the Last Glacial Period, reaching their lowest levels around the Last Glacial Maximum approximately 25 000 years ago. A notable population expansion occurred during the warmer intervals around 10 000–12 500 years ago, following the retreat of the ice sheets. Additionally, a further decline in *P. nigra* was observed during the period of 100–200 generations ago, or approximately 2500–5000 years ago under the same generation time assumption. This coincides with the emergence of the earliest human civilizations up to the Roman Empire in the Mediterranean basin, as well as a sea level rise of approximately 2.5 m between 1500 BC and the dawn of the Common Era (Yasur‐Landau et al., 2021).

To assess the divergence time and gene flow between *P. banksiana* and *P. contorta*, we employed fastsimcoal2 (Excoffier et al., 2013, 2021), which utilizes the joint site frequency spectrum (SFS) based on a coalescent simulation approach. Among the five tested models, the best‐fitting model suggested varying gene flow rates over time, following the divergence of the two species (Figure 2B). Specifically, the model posited that *P. banksiana* and *P. contorta* diverged from their common ancestor approximately 580 000 generations ago, with a decrease in gene flow rate occurring around 250 000 generations ago. Under the assumption of a 25‐year generation time (Brown et al., 2004), this divergence corresponds to an estimated time of around 14.4 million years ago, coinciding with the later stage of the Middle Miocene climate transition (Flower & Kennett, 1994; Pound et al., 2012). Notably, the gene flow rate transitioned and decreased around 6.3 million years ago, coinciding with the North American prairie expansion during the late Miocene (Retallack, 1997, 2001), where a cool and dry climate, coupled with the expansion of tall C4 grasslands, likely impeded gene flow between the two species. The simulations demonstrated significant asymmetry in introgression between the two lineages during the early phase of divergence, with significantly higher rates of gene flow from *P. contorta* to *P. banksiana* that later reversed direction. Parameter estimates for divergence time, gene flow rate, timing of gene flow rate changes, *N*_e_, and their corresponding 95% confidence intervals are listed in Table S4.

### Estimation of genome‐wide LD and recombination rate

Previous studies on LD decay in conifers typically relied on short gene fragments, which may have introduced bias due to genomic heterogeneity (Larsson et al., 2013; Pyhäjärvi et al., 2011). To gain a comprehensive genome‐wide understanding of LD levels, we analyzed LD decay using whole‐genome SNP data. Genome‐wide LD analysis for all SNP pairs within a maximum distance of 100 kb revealed that LD decays rapidly over short genomic distances, consistent with previous results based on short gene fragments (Figure S6) (Brown et al., 2004; Lu et al., 2016; Pyhäjärvi et al., 2007).

The LD patterns are closely related to the recombination rate, which can be indirectly inferred from LD. Mutations occur on specific haplotypes, resulting in associations that arise solely from the mutational process, and these associations decay as the recombination rate increases. Consequently, regions with higher recombination rates typically exhibit lower LD.

To further investigate the variation in LD and the recombination rate at a finer scale, and to assess how these factors influence different modes of selection, we estimated the population‐scaled recombination rate (*ρ*) across the genome. The genome‐wide mean *ρ* was highest in *P. nigra* (*ρ* = 0.039), followed by *P. contorta* (*ρ* = 0.028) and *P. banksiana* (*ρ* = 0.023). When large genomic segments (>100 kb) with a constant *ρ* were removed, the mean *ρ* values were found to be 0.016, 0.013, and 0.011 for *P. nigra*, *P. contorta*, and *P. banksiana*, respectively.

**Figure 3:**
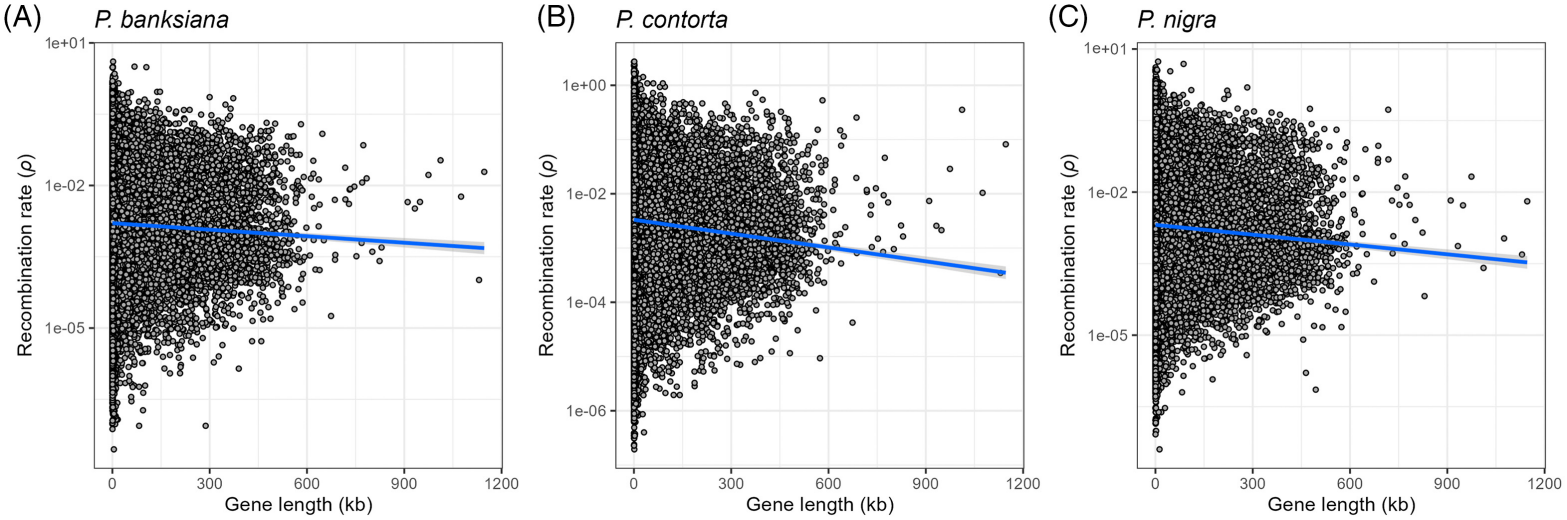
Correlations between population‐scaled recombination rate (*ρ*) and gene length among three *Pinus* species. Each dot represents an annotated gene of the reference genome. (A–C) *P*‐value <2.6e‐15.

To examine the relationships between recombination and various genomic features, we recalculated *ρ* across different window sizes, excluding windows containing fewer than 10 SNPs. Genome‐wide *ρ* patterns based on 1‐ and 10‐Mb windows revealed relatively low recombination rates near chromosome centers and ends, with elevated rates in intermediate regions (Figures S7 and S8). Moderate positive correlations in *ρ* were observed among the three species (Figure S9). Further analyses showed significant positive correlations between *ρ* and both *π* and gene density (Figures S10 and S11), whereas *ρ* was negatively correlated with gene length and cumulative gene length across both window scales (Figure 3; Figure S12).

To clarify the basis of the negative relationship between *ρ* and gene length, we quantified cumulative exon and intron lengths for each window. Recombination rate (*ρ*) was negatively correlated with cumulated intron length but showed a slight positive correlation with exon length, indicating that the overall negative correlation between *ρ* and gene length is primarily driven by intron content (Figures S13 and S14). The cumulated exon length within genomic windows is also strongly influenced by local gene density, which itself is positively correlated with recombination rate. We therefore interpret the weak positive correlation between *ρ* and cumulated exon length as an indirect effect driven by gene density rather than by exon length *per se*. In addition, this positive correlation is weak and not consistently significant across species (Figure S13), whereas the negative correlation between *ρ* and cumulated intron length is robust and significant across window sizes and species (Figure S14).

To assess the role of TEs in the negative correlation between *ρ* and gene length, we calculated the TE‐covered length of genes and TE density within genomic windows. The results indicate that *ρ* is negatively correlated with both the TE‐covered length of genes and TE density in genomic windows (Figure S15).

**Figure 4:**
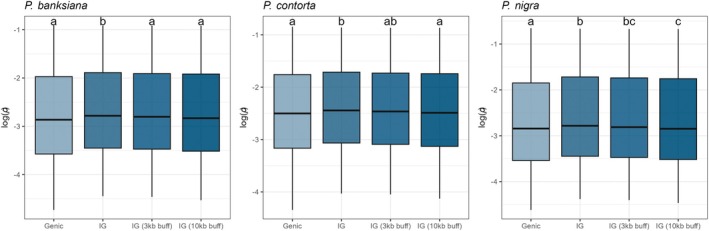
Recombination rates (*ρ*) vary between genic and intergenic regions. To remove the effect of 5′ and 3′ regulatory regions at the proximity of genes, buffered intergenic regions were defined by excluding 3‐ and 10‐kb flanking regions upstream and downstream of genes. Different letters indicate statistically significant differences among groups based on Tukey‐adjusted pairwise comparisons of estimated marginal means (*α* = 0.05). IG, intergenic regions.

To assess variation in *ρ* between functional genomic regions, we compared genic and intergenic recombination rates. Because gene‐flanking regions often contain promoters and cis‐regulatory elements, intergenic regions excluding 3‐ and 10‐kb windows upstream and downstream of genes were also analyzed. The upper and lower 2.5% of values were removed to limit the influence of outliers. The results indicated that the mean *ρ* was lower in genic than in intergenic regions (Figure 4). However, intergenic *ρ* decreases when regions close to genes are excluded, suggesting that recombination decreases with distance from genes.

### Correlations of the genomic landscapes of differentiation and diversity

To investigate the evolutionary processes shaping the genomic landscape in pines, we estimated genomic features including genetic differentiation (or relative genetic divergence, *F*_ST_), between‐population diversity (or absolute genetic divergence, *d*_XY_), and within‐population nucleotide diversity (*π*) across 10‐kb non‐overlapping windows. To reduce the uncertainty and potential bias, genomic windows containing fewer than 10 SNPs were excluded. A total of 498 817, 499 040, and 499 040 windows were analyzed for the species pairs *P. banksiana*/*P. contorta*, *P. nigra*/*P. banksiana*, and *P. nigra*/*P. contorta*, respectively. The genome‐wide average *F*_ST_ values for these species pairs were 0.308, 0.601, and 0.570 (Figure S16A), with corresponding average *d*_XY_ values of 0.0050, 0.0132, and 0.0128 (Figure S16B).

**Figure 5:**
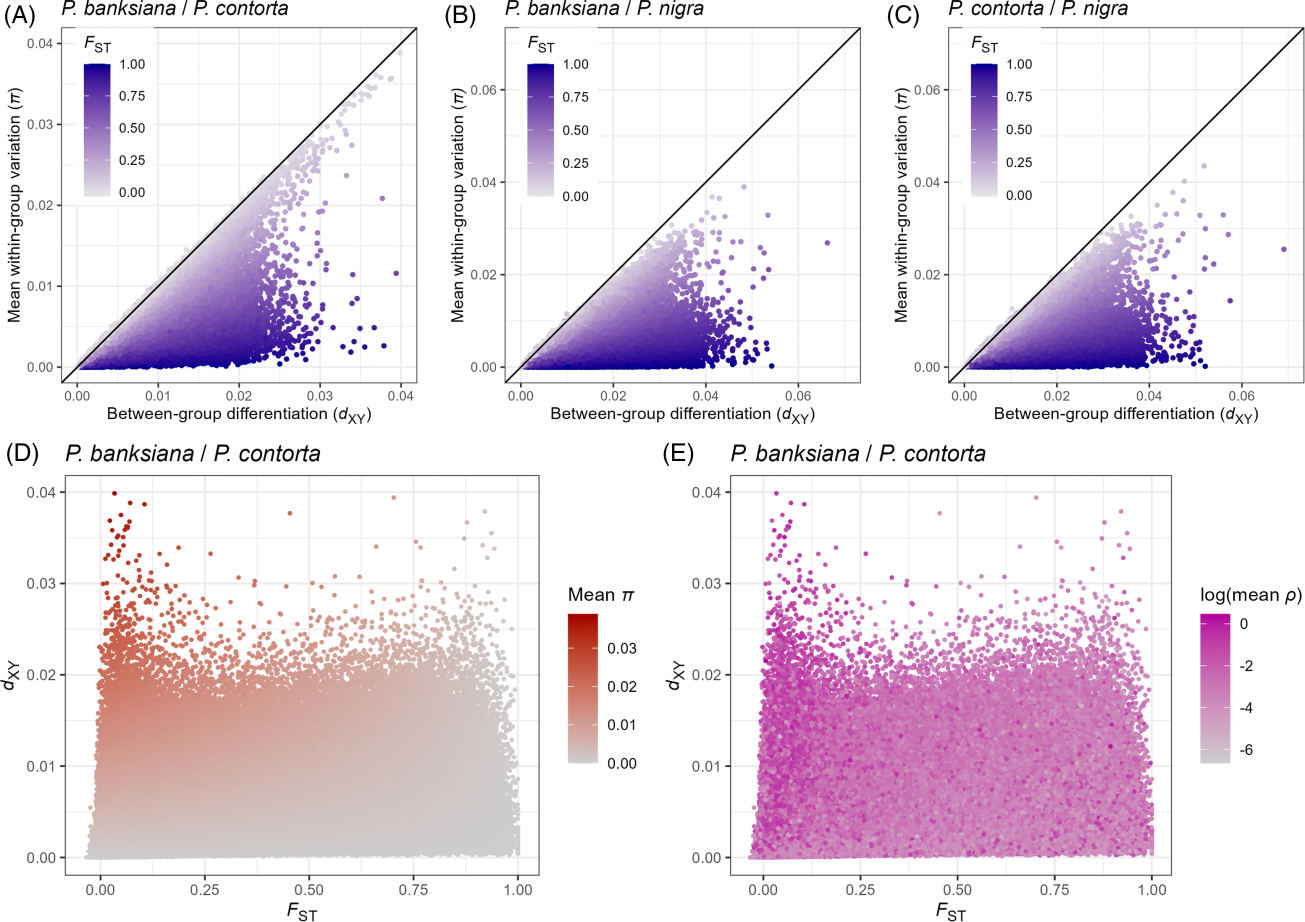
Genome‐wide analysis of nucleotide diversity, divergence, and recombination in three *Pinus* species pairs. Each dot represents a non‐overlapping 10‐kb genomic window. (A–C) Scatter plots showing the distribution of genomic windows with respect to within‐population nucleotide diversity (*π*) and between‐population nucleotide diversity (*d*_XY_) of the species pairs *P. banksiana*/*P. contorta*, *P. banksiana*/*P. nigra*, and *P. contorta*/*P. nigra*, respectively. The color of each dot reflects the level of relative genetic divergence (*F*_ST_). The black lines in (A–C) indicate the line of equality between *π* and *d*_XY_. (D) Scatter plot illustrating the relationship between *d*_XY_ and *F*_ST_ for the *P. banksiana*/*P. contorta* species pair, with dots colored by levels of *π*. (E) Scatter plot of the same variables, with dots colored by population‐scaled recombination rate (*ρ*).

Across all three species pairs, most genomic regions showed lower *π* than *d*_XY_ (Figure 5A−C). These patterns reflect heterogeneous genomic divergence, as *π* is expected to be equal to *d*_XY_ in undifferentiated populations. Regions with lower *F*_ST_ values displayed comparable *π* and *d*_XY_ values, while those with high *F*_ST_ values showed lower *π*/*d*_XY_ ratios. This pattern was most pronounced between *P. banksiana* and *P. contorta* and less evident in comparisons involving *P. nigra*, likely reflecting their longer divergence time.

We next examined the relationship between *F*_ST_ and *d*_XY_, focusing on the *P. banksiana*/*P. contorta* pair, as *F*_ST_ values for the *P. nigra* comparisons were largely saturated near 1 (Figure S16). The plot of *F*_ST_ against *d*_XY_ showed that genomic windows with either high or low *F*_ST_ exhibited a broader range of *d*_XY_ than those with intermediate *F*_ST_ values (Figure 5D). Windows with low *F*_ST_ and high *d*_XY_ were associated with higher *π* and *ρ*, although the correlation with *ρ* was weaker and less consistent than that with *π* (Figure 5D,E).

To characterize patterns of genomic differentiation, we identified putative genomic islands and valleys based on variation in *F*_ST_, *d*_XY_, and *π*, and then calculated their average window size and *ρ* for regions representing different selective scenarios (Table 2). Three main evolutionary models were considered for genomic islands: divergence with gene flow, allopatric selection, and recurrent selection (Cruickshank & Hahn, 2014; Glover et al., 2024; Han et al., 2017; Irwin et al., 2018). These scenarios are characterized by reduced *π* and elevated *F*_ST_, but differ in *d*_XY_ levels of high, intermediate, and low, respectively, reflecting distinct selective processes.

**Table 2:** Summary of 10‐kb non‐overlapping genomic windows that fit different scenarios of divergence

Scenario	Windows number	Mean window size^a^	Mean rho^b^	Mean Tajima's *D* in *P. banksiana* ^b^	Mean Tajima's *D* in *P. contorta* ^b^	Mean |iHS| in *P. banksiana* ^b^	Mean |iHS| in *P. contorta* ^b^
Genome‐wide	498 817		0.010	0.225	−0.082	0.304	0.348
Divergence with gene flow	13	1.077 (1.294)***	0.013	−1.237**	−1.336**	0.594	0.440
Allopatric selection	3597	1.322 (1.294)***	0.007	−0.954***	−0.834***	0.357**	0.409***
Recurrent selection	371	1.032 (1.293)**	0.005**	−0.904***	−0.735***	0.368	0.501**
Balancing selection	1003	1.026 (1.091)	0.079***	0.709***	0.469***	0.258**	0.382**
Background/parallel selection	2878	1.015 (1.015)	0.012*	−1.014***	−1.096***	0.463***	0.653***

^a^ Values in parenthesis indicate the mean of randomized null distribution. Asterisks indicate values significantly different from the randomized distribution (two‐sided permutation test, *P < 0.05; **P < 0.01, ***P < 0.001).

^b^ Asterisks indicate significant differences between the group and 3600 randomly selected windows from background in two‐tailed Welch's *t*‐test (*P < 0.05, **P < 0.001, ***P < 2.2 × 10^−16^).

For genomic valleys, two scenarios were examined: balancing selection and a combined background/parallel selection model, which may also encompass introgression (Charlesworth, 2006; Cvijović et al., 2018; Roesti et al., 2012, 2014; Zhang et al., 2023). Both are characterized by reduced *F*_ST_; however, balancing selection is associated with elevated *π* and *d*_XY_, whereas background/parallel selection shows reductions in both metrics.

Among the genomic regions assigned to specific evolutionary models, 45.8% corresponded to allopatric selection, followed by 36.6% fitting background/parallel selection, 12.8% balancing selection, 4.7% recurrent selection, and 0.2% to divergence with gene flow (Table 2). Regions associated with allopatric selection showed significantly larger average window sizes than expected under the randomized null distribution, with three extending up to 120 kb, whereas regions associated with divergence with gene flow and recurrent selection were significantly smaller than expected. In contrast, regions associated with genomic valleys showed no significant difference from the randomized distribution. Recombination rates (*ρ*) in putative genomic valleys were significantly higher than the background level, particularly in regions under balancing selection. In contrast, putative genomic islands associated with allopatric selection and recurrent selection exhibited lower *ρ* values, although the reduction was not statistically significant in the former.

To further validate the inferred selection patterns, we calculated Tajima's *D* for all genomic regions (Table 2; Figure S17A,B), a statistic that measures deviations from neutrality (Tajima, 1989). Genomic regions consistent with the balancing selection scenario showed positive average Tajima's *D* values exceeding background levels in both species, whereas regions associated with other selective regimes displayed negative average Tajima's *D* values. These contrasting patterns are consistent with theoretical expectations, where positive Tajima's *D* indicates an excess of intermediate‐frequency alleles typically produced by balancing selection, and negative values reflect an excess of rare alleles often associated with directional or purifying selection.

Because deviations in Tajima's *D* can also result from demographic changes or introgression, we additionally calculated the absolute integrated haplotype statistics (|iHS|) to detect unusually long haplotypes indicative of selective sweeps (Table 2; Figure S17C,D). The iHS statistic compares haplotypes within genomic regions while accounting for local recombination rates, making it particularly effective for identifying recent selection (Voight et al., 2006). Across the genomic regions classified under specific evolutionary models, all scenarios exhibited significantly higher |iHS| values than the genomic background, except the divergence‐with‐gene‐flow scenario in both species and recurrent selection and balancing selection scenarios in *P. banksiana*. Although |iHS| was elevated in the divergence‐with‐gene‐flow regions of both species, the lack of statistical significance likely reflects limited data and power. Moreover, both Tajima's *D* and |iHS| analyses revealed some windows with extreme values outside the five scenarios (Figure S17), indicating that other selective processes may also shape parts of the genome landscape beyond the genomic islands and valleys.

## DISCUSSION

Comparative genomic studies in flowering plants have revealed substantial insights into how genomic landscapes are sculpted, yet the evolutionary forces shaping genomic islands and valleys in conifers and their interaction with recombination have remained poorly understood. By analyzing whole‐genome data from three pine species, we show that the conifer genomic landscape is strongly influenced by pronounced heterogeneity in recombination rates, with reduced recombination within unusually long introns contributing to the evolution of exceptionally long genes. Our results further indicate that while allopatric selection is the dominant driver of genomic islands and species divergence, conserved genomic valleys are maintained through balancing, parallel, and background selection. Together, these findings underscore the importance of the interplay between selective pressures and local recombination environments in shaping genomic diversity and structure in long‐lived, non‐flowering plant lineages.

### Infraspecific classification, divergence timing, and demographic changes

The clustering analyses support the classification of *P. nigra* subspecies (Figure 1; Figure S5). In contrast, although morphological variations have led some taxonomists to recognize distinct subspecies of *P. contorta*, our results do not support the classification, except for *P. contorta* ssp. *contorta* (Figure 1; Figure S5). This result is consistent with previous studies concerning the genetic base of the infraspecific classification of *P. contorta* (Fazekas & Yeh, 2006; Hernández‐León et al., 2013; Wheeler & Guries, 1982). The discrepancy between genetic data and morphology‐based classification of these groups may indicate that they diverged relatively recently. This could imply that lineage sorting is incomplete, and there might be ongoing gene flow between these groups. It is important to note, however, that our sampling did not cover the entire distribution range for these two species, highlighting the need for more comprehensive sampling in future studies to achieve a more rigorous infraspecific classification.

The Sequentially Markovian coalescent‐based analyses of demographic dynamics indicate that the Last Glacial Period has significant impact on all three pine species (Figure 2A). These results are consistent with previous research using organelle markers and fossil evidence (Godbout et al., 2005; Macdonald & Cwynar, 1985, 1991; Marshall et al., 2002; Mcleod & Macdonald, 1997; Roiron et al., 2013; Rudolph & Yeatman, 1982). Interestingly, we observed a further decline in *P. nigra* approximately 2500–5000 years ago, which may be associated with human impacts beginning in the Iron Age (Rodrigo et al., 2004) or may reflect a contraction linked to the period of intraspecific lineage splitting (Scotti‐Saintagne et al., 2019).

Our estimated divergence timeframe based on simulations aligns the MCMCTree analyses utilizing a coding sequence dataset with fossil calibration (Jin et al., 2021). The asymmetrical introgression from *P. contorta* to *P. banksiana* was previously identified through organelle DNA analyses by Godbout et al. (2012), who proposed that these events occurred more recently than suggested by our findings. Interestingly, both biological and genetic evidence suggest that introgressed loci are often associated with disease and pest resistance from *P. banksiana to P. contorta* (Cullingham et al., 2013; Godbout et al., 2005; Wu et al., 1996), although the exact timing of these introgression events warrants further investigation. Identifying these introgressed loci in future research will be particularly valuable for breeding efforts.

We would like to point out that the gene flow during the early stage of divergence in our model does not imply sympatric or parapatric speciation. Rather, it likely reflects intermittent secondary contact associated with glacial–interglacial cycles. The observed asymmetric introgression can be explained by the early arrival of *P. contorta* in the contact zone before *P. banksiana*, resulting in gene transfer from the locally abundant species toward the invasive one (Currat et al., 2008; Du et al., 2011).

### Recombination rates in pines

LD‐based methods for estimating recombination rates rely on several simplifying assumptions about the studied populations (Peñalba & Wolf, 2020; Samuk & Noor, 2022). For example, it is often assumed that demographic processes that can distort genome‐wide LD patterns have not occurred (Auton & Mcvean, 2007). Simulation studies have demonstrated that demographic events, such as population size change and gene flow, can reduce the accuracy and power of LD‐based recombination rate inferences (Dapper & Payseur, 2017; Samuk & Noor, 2022). Continuous gene flow could lead to overestimation of the recombination rates, as might be the case in this study. Future efforts that refine SNP calling accuracy, incorporate proper demographic models for *N*_e_ estimation, and integrate detailed minor allele frequency (MAF) information with high‐resolution linkage maps will be crucial for advancing a comprehensive understanding of recombination dynamics and interspecific comparisons in pines.

Previous studies in angiosperms have found positive correlations between recombination rates, nucleotide diversity, and gene density (Brazier & Glémin, 2022; Stapley et al., 2017; Tiley & Burleigh, 2015), but data on conifers remain sparse. Our genome‐wide study confirmed these correlations among the three examined pine species (Figures S10 and S11). Additionally, we observed a negative correlation between recombination rates and gene length (Figure 3; Figure S12). This negative correlation appears to be predominantly influenced by introns rather than exons in these species (Figures S13 and S14), aligning with findings from previous studies, which showed a negative correlation between intron length and recombination rates in various non‐gymnosperm species, including *Arabidopsis*, *Drosophila*, and humans (Carvalho & Clark, 1999; Comeron & Kreitman, 2000; Wright et al., 2003).

Besides, gymnosperm genomes are characterized by longer genes and introns, as well as a high prevalence of TEs within introns (Liu et al., 2021, 2022; Niu et al., 2022; Nystedt et al., 2013; Stival Sena et al., 2014; Wegrzyn et al., 2014). The presence of these TEs likely contributes significantly to the observed increases in total intron length. We also observed a negative correlation between TE density and recombination rate (Figure S15), consistent with patterns reported across diverse species (Kent et al., 2017). Furthermore, gene length may directly influence recombination rates because recombination hotspots are often localized near the 5′ and 3′ regions, leading to a reduction in average recombination rate as the gene length increases (Brazier & Glémin, 2024). Given the limited number of studies on recombination rates in conifers, further investigation of fine‐scale recombination rate patterns using well‐annotated reference genomes for each species is essential to advance our understanding of these genomic features in conifers.

This prevalent negative relationship between TE density and recombination rate is often interpreted as the combined outcome of selection and genome defense mechanisms, where low‐recombination regions facilitate TE accumulation due to reduced efficacy of purifying selection; alternatively, TE accumulation itself may contribute to recombination suppression (Choi & Lee, 2020; Kent et al., 2017). Because TE‐related SNPs were retained in our dataset, it is possible that these regions contribute substantially to the significant negative correlation observed between accumulated intron length and the recombination rate. DNA methylation is widely recognized as a key regulatory mechanism suppressing TE activity and influences local chromatin structure, thereby potentially modulating recombination landscapes. Although methylation data were not available in this study, integrating epigenetic profiles would provide critical insight into whether TE‐rich, low‐recombination regions in pines are also characterized by elevated methylation levels. Future work incorporating methylation data will therefore be essential to disentangle the interplay among TE accumulation, recombination suppression, and epigenetic regulation in shaping the large and complex genomes of conifers.

Interpretation of interspecific differences in recombination rate should be made with caution, as our estimates rely on reference‐based mapping to the *P. tabuliformis* genome. Differences in genetic divergence among species result in variable mapping efficiency, with more divergent species exhibiting reduced mapping rate and coverage of the genome (Table S1). This can systematically bias SNP discovery toward more conserved regions (Table S2), leading to differences in the accuracy and comparability of *ρ* estimates across species. For example, missing data in highly polymorphic or structurally variable regions may cause overestimation of recombination rates, as structural variants such as inversions are typically associated with reduced recombination (Ortiz‐Barrientos et al., 2016). Therefore, while our results provide robust insights into relative recombination patterns within species and into genome‐wide recombination landscapes, comparisons of recombination rate across species should be interpreted cautiously, as they may be influenced by reference bias.

### Genomic differentiation, selection, and the role of genomic islands in evolutionary history

In our study, we observed widespread genomic differentiation and conservation between *P. banksiana* and *P. contorta*, suggesting a heterogenous genomic landscape shaped by both natural selection, reproductive isolation, and recombination rate (Table 2). However, genomic landscapes are often modulated by multiple types of selection, which makes the identification of predominant processes in selection scenarios more indicative rather than definitive conclusions. We also found that genomic features such as *π*, *ρ*, and Tajima's *D* values are correlated with both *d*_XY_ and *F*_ST_, as well as with their interaction (Figure 5; Figure S17). This finding may explain the weak collinearity often observed in previous studies when comparing a single genomic feature with only one divergence parameter (*d*_XY_ or *F*_ST_).

We found that the allopatric selection model is the primary mechanism contributing to the formation of genomic islands in *P. banksiana* and *P. contorta*, consistent with findings from other perennial tree species, such as *Populus* and *Castanopsis* (Chen et al., 2024; Shang et al., 2023). Interestingly, we found that genomic islands associated with allopatric selection tend to have a larger average size, whereas those associated with gene flow and recurrent selection tend to be smaller (Table 2). The question of whether genomic islands and valleys should expand or contract over time is particularly relevant in the context of ongoing divergence. Theoretical models predict that genomic islands should enlarge as populations undergo divergence, driven by the process of divergence hitchhiking (Smadja et al., 2008; Via & West, 2008), while this could vary under different divergence models (Nosil et al., 2009). Several empirical studies have failed to demonstrate a positive correlation between divergence time and average genomic island size across avian and insect species (Han et al., 2017; Riesch et al., 2017; Sendell‐Price et al., 2020), which may be influenced by the confounding effects of different types of genomic islands. Our findings indicate that the sizes of genomic islands and valleys could vary based on their formation processes and evolutionary history. Therefore, we emphasize the importance of considering the evolutionary history of these genomic islands and valleys to better understand how time and gene flow shape their size. However, the allopatric model predicts smaller genomic islands in comparison with models incorporating gene flow (Nosil et al., 2009), which contradicts our observation. The reasons why genomic islands associated with allopatric selection in pines tend to be larger, whereas those associated with gene flow and recurrent selection tend to be smaller, remain unclear.

Recombination rate is an important factor related to genomic islands and valleys. We observed that highly diverged regions tend to exhibit low *ρ*, consistent with the theory and observations, supporting that reduced recombination is linked to the maintenance of divergent genomic features (Burri et al., 2015; Renaut et al., 2013). In the case of genomic islands driven by selection, the extent of the hitchhiking effect can be influenced by both the recombination rate and the intensity of selection. Theoretically, the linkage associated with the hitchhiking effect is wider in low‐recombination regions, leading to a more significant reduction in diversity across larger chromosome segments. However, our findings indicate that while the average *ρ* in genomic regions subject to recurrent selection was lower than the background (Table 2), the size of these regions did not appear to be larger when compared to those fitting allopatric selection scenarios.

### Selection mechanisms maintaining conserved genomic regions

Genomic valleys have received significantly less attention compared with genomic islands. One possible explanation for this disparity is that loci found within these valleys rarely play a direct role in speciation. Additionally, genomic valleys can primarily be identified in well‐diverged genomes of taxa or populations, while genome scanning studies tend to concentrate on the early stages of divergence that may be linked to speciation. However, identifying these regions enhances our understanding of the long‐term effects of divergence on the genomic landscape. For instance, the left‐skewed distribution of extreme *F*_ST_ values observed during later stages of divergence, such as in the *P. banksiana*/*P. nigra* and *P. contorta*/*P. nigra* species pairs from our study, likely reflects the combined influences of various types of selection and adaptive introgression (Figure S16B).

Although genomic valleys characterized by low *d*_XY_ and *F*_ST_ can arise from both background selection and parallel selection, the impact of parallel selection is likely small because the probability and extent of parallelism generally decline as divergence time increases between taxa (Arendt & Reznick, 2008, Conte et al., 2012). Even if parallel selection occurred during the early stages of divergence between *P. banksiana* and *P. contorta*, such genomic valleys would not be maintained over long divergence time unless they were continually subjected to strong background selection.

Genomic regions associated with balancing selection demonstrated significantly higher average *ρ*, supporting the hypothesis that balancing selection may influence recombination dynamics (Schield et al., 2022). Notably, Tajima's *D* and |iHS| values in regions associated with balancing selection and parallel/background selection are both significantly deviated from the background value, indicating that few detected valleys resulted from incomplete lineage sorting, as genomic valleys arising from this phenomenon are expected to exhibit neutrality. This result is expected, as the long divergence history between *P. banksiana* and *P. contorta* suggests that genomic valleys caused by incomplete lineage sorting should have substantially diminished over time (Charlesworth, 2006).

## MATERIALS AND METHODS

### Sample collection, DNA extraction, and whole‐genome sequencing

In our previous study, we detailed the collection of a total of 100 samples from three pine species (Estravis Barcala et al., 2024). In brief, we collected vegetative buds from 37 *P. banksiana*, 29 *P. contorta*, and 34 *P. nigra* individuals, sourced from a genus‐wide experiment conducted at the Arboretum Sofronka in the Czech Republic (Kaňák, 2016). These samples correspond to 7, 21, and 13 distinct wild collecting sites, respectively (Figure S1; Table S1). The distribution information of the North American and European species was retrieved and modified from Wilson et al. (2013) and Caudullo et al. (2017), respectively. Genomic DNA was extracted from bud tissue utilizing the E.Z.N.A. SP Plant DNA Kit (Omega Bio‐Tek, Norcross, GA, USA), following the manufacturer's protocols. Following extraction, the quality‐controlled DNA samples were dispatched for sequencing. The sequencing process for all samples was executed by Annoroad Gene Technology (Beijing, China) on the DNBSEQ‐T7 platform, employing a paired‐end read length of 150 bp for comprehensive whole‐genome sequencing, resulting in 277–530 billion raw sequences for each sample (Table S1). The quality of the per‐base sequences was evaluated using FastQC v0.11.9 (https://www.bioinformatics.babraham.ac.uk/projects/fastqc/ [Accessed November 18, 2025]) and MultiQC v1.12 (Ewels et al., 2016). Adapter sequences were eliminated from the reads, along with consecutive stretches of low‐quality bases (quality score <20) and ambiguous bases (Ns) at both the 5′ and 3′ ends, using AdapterRemoval v2.3.1 (Schubert et al., 2016). Reads shorter than 25 bp were discarded.

### Mapping, variant calling, and filtering

In preparation for mapping, the reference genome, *P. tabuliformis* genome v1.0 (Niu et al., 2022), was partitioned into six subsets, with each chromosome further divided into 3–5 segments, due to the size limitations inherent in many analytical tools. Trimmed paired‐end reads from each sample were aligned to the reference genome using Bowtie2 v2.4.5, employing the options ‘‐‐no‐unal’ and ‘‐‐very‐sensitive’ (Langmead & Salzberg, 2012). The aligned reads were subsequently compressed, sorted, and duplicate PCR reads were removed using Sambamba v0.7.1 (Tarasov et al., 2015).

Variant calling was performed using GATK v4.3.0.0, employing the HaplotypeCaller for all‐site calling, CombineGVCFs for merging multi‐sample records, and GenotypeGVCFs for genotyping (Mckenna et al., 2010). Repetitive elements and TEs were not masked during the variant calling process to ensure maximum data retention for subsequent analyses. Variants and invariant sites were subjected to separate filtering processes. Initial filtering of raw variants and invariants was conducted using BCFtools v1.17 (Danecek et al., 2021). For variants, we applied generic hard‐filtering criteria, excluding variants with the following characteristics: FS >60.0, SOR >3.0, MQ <20.0, QD <2.0, MQRankSum <−12.5, or ReadPosRankSum <−8.0. Additionally, variants with mapping depths exceeding three times the average or below one‐third of the average were filtered out. Single genotypes with read depths below 3 or genotype quality scores below 10 were designated as missing. Furthermore, SNPs located within 5 base pairs of indels, monomorphic SNPs, or those with more than 20% missing genotypes or indels were eliminated. SNPs exhibiting MAF below 0.01 were also excluded. Multi‐allelic SNPs were removed unless they were suitable for subsequent analyses.

For invariant sites, the missing genotypes initially set to ‘0’ by GATK HaplotypeCaller were replaced with ‘.’ Invariants with mapping depths exceeding three times the average or below one‐third of the average, as well as genotypes with more than 20% missing data, were removed. The counts of private and shared SNPs for each species and their combination were determined using ‘isec’ function in BCFtools.

### Intraspecies genomic diversity and interspecies genomic divergence

To evaluate and compare genetic diversity among the three pine species, we calculated *π*, *d*_XY_, and *F*_ST_, incorporating both variant and invariant sites. This analysis was conducted using *pixy* v1.2.5.beta1 (Korunes & Samuk, 2021). Additionally, Tajima's *D* statistics were calculated utilizing VCFtools v0.1.16 (Danecek et al., 2011). Each of the statistical measures was executed across both 10‐ and 100‐kb non‐overlapping windows. Furthermore, nucleotide diversity was assessed for various categories of functional elements, including zerofold degenerate coding sites, fourfold degenerate coding sites, introns, 3′ UTRs, 5′ UTRs, and intergenic regions in all three pine species. Zerofold and fourfold degenerate coding sites were identified and isolated based on the *P. tabuliformis* genome v1.0 annotation, utilizing the ‘get_degeneracy.py’ script (https://github.com/zhangrengang/degeneracy/ [Accessed November 18, 2025]).

To compare our nucleotide diversity results with those reported by Zhao et al. (2024), we retrieved raw sequencing data for 10 *P. nigra* samples from the National Center for Biotechnology Information. The downloaded sequences underwent trimming, mapping, variant calling, and filtering using the aforementioned methods, with the exception that filtering was conducted using a minor allele count threshold of ≤2 instead of a MAF threshold of ≤0.01 due to differences in sample sizes. Subsequent estimates of nucleotide diversity were conducted using the previously described methods.

### Population structure and clustering analysis

For the analysis of population structure, we excluded highly correlated SNPs by performing LD‐based SNP pruning using PLINK v1.90 beta 4.9 (Chang et al., 2015). A sliding window of 50 bp with a step size of 10 bp was employed to scan and eliminate pairs of SNPs exhibiting a squared correlation (*r* ^2^) greater than 0.2 within the window. The pruned SNPs were then subsequently utilized for PCA in PLINK v1.90 beta 4.9. To further investigate the population structure across all individuals, we employed ADMIXTURE v1.3.0, varying *K* from 2 to 10 (Alexander et al., 2009). The optimal value of *K* was determined based on cross‐validation error.

The sample NIG_J84_1, initially labeled as *P. nigra* ssp. *laricio*, was identified as a mislabeling of *P. nigra* ssp. *nigra* through PCA and clustering analyses. Consequently, the label was corrected for subsequent analyses.

### Demographic history

The population size variation for the three species through time was inferred using SMC++ v1.15.4 (Terhorst et al., 2017). Runs of homozygosity exceeding 50 000 bp were categorized as missing. The mutation rate was set as 1.9 × 10^−8^ per site per generation (Brown et al., 2004; Gao et al., 2012).

The demographic histories associated with speciation of *P. banksiana* and *P. contorta*, as well as potential scenarios influencing their current genetic structure, were inferred through a coalescent simulation‐based method employing fastsimcoal2 v2.7.93 (Excoffier et al., 2013, 2021). Biallelic SNPs were used without MAF filtering for estimating unfolded two‐dimensional SFS (2D‐SFS) using ANGSD v0.940 (Korneliussen et al., 2014). The analysis incorporated five relatively straightforward models that focused on gene flow following divergence: no gene flow, continuous gene flow, early gene flow only, recent gene flow only, and a distinct gene flow matrix after divergence. For each model, 100 000 coalescent simulations were run to estimate the expected 2D‐SFS and the log‐likelihood of the demographic parameters associated with each model. Monomorphic sites and entries in the observed SFS entries with counts fewer than 10 were excluded from parameter inference. Global maximum likelihood estimates were obtained from 100 independent runs, each consisting of 50 cycles of the Expectation‐Conditional Maximization algorithm. The optimal run for each model was determined based on the maximum likelihood value across the 100 independent runs, and the best‐fitting model was selected according to Akaike's information criterion.

Confidence intervals for the parameters of the best model were calculated through parametric bootstrapping, which involved 100 bootstrap estimates of the 2D‐SFS generated via pseudo‐sampling with site replacement in ANGSD (Korneliussen et al., 2014). For each bootstrap, 100 independent runs were conducted using the initial parameter values derived from the best run based on the true 2D‐SFS. The parameter estimates from the best run for each bootstrap replicate were subsequently used to ascertain the 95% confidence intervals.

### LD and population‐scaled recombination rate

The decay of LD for each species was estimated for all pairs of SNPs in a maximum distance of 100 kb using PopLDdecay v3.42 (Zhang et al., 2018). To estimate the *ρ*, phased VCF files and inferred ancestral allelic state were prepared. The VCF files were phased for each species using SHAPEIT4 v4.2.2 (Delaneau et al., 2019), while the ancestral allelic state was inferred using est‐sfs v2.04 (Keightley & Jackson, 2018) with two outgroups and the Kimura 2‐parameter model. For *P. banksiana* and *P. contorta*, the nucleotide states of the first outgroup were assigned based on the 70% majority states observed in samples of another species, and the nucleotide states of the second outgroup were assigned according to the 70% majority states of *P. nigra* samples. For *P. nigra*, the states of the first outgroup were assigned to the nucleotide states of *P. tabuliformis* reference genome, and the states of the second outgroup were allocated based on the 70% majority states derived from the combined samples of *P. banksiana* and *P. contorta*.

Forty randomly selected haplotypes were utilized to estimate *ρ* using LDhelmet v1.10 (Chan et al., 2012). The workflow and parameters were configured in accordance with the recommendations provided in the manual. Finally, the mean *ρ* value for each block generated by LDhelmet was employed for subsequent analyses.

### Local genomic signatures across the genomic landscape

The genomic landscape of the three species was investigated by analyzing various genomic features, including *F*_ST_, *d*_XY_, *π*, *ρ*, and Tajima's *D* statistics, within 10‐kb non‐overlapping windows. Windows containing fewer than 10 SNPs were excluded. Consequently, a total of 498 817 windows were retained and subsequently classified into distinct scenarios of genomic divergence based on their *F*_ST_, *d*_XY_, and *π* values. Windows with extreme *F*_ST_ values, accompanied by their *d*_XY_ and *π* values, were used for inferring the genomic islands and valleys.

Initially, *F*_ST_ values corresponding to the upper 5% and lower 5% thresholds were used to identify candidate genomic islands and valleys, respectively. Subsequently, the adjacent candidate windows were merged and their *d*_XY_, *π*, *ρ*, and Tajima's *D* were recalculated. Finally, the upper 5% and lower 5% thresholds of *d*_XY_ and *π*, derived from the non‐merged dataset, were employed to categorize the windows from the merged dataset into five distinct scenarios of post‐divergence, as outlined by previous studies (Charlesworth, 2006; Cvijović et al., 2018; Han et al., 2017; Irwin et al., 2018; Roesti et al., 2014).

In brief, five evolutionary scenarios are proposed: (1) divergence with gene flow, with *F*_ST_ ≥95%, *d*_XY_ ≥95%, and *π* ≤5%, consistent with local adaptation and reduced gene flow at selected loci; (2) allopatric selection, with *F*_ST_ ≥95%, *d*_XY_ between 5 and 95%, and *π* ≤5%, reflecting within‐population selection without gene flow; (3) recurrent selection, with *F*_ST_ ≥95%, *d*_XY_ ≤5%, and *π* ≤5%, where low *d*_XY_ and *π* indicate shared intense selection before divergence and recent selection after divergence; (4) balancing selection, with *F*_ST_ ≤5%, *d*_XY_ >95%, and *π* ≥95%, referring to the long‐term maintenance of polymorphism; and (5) background selection, parallel selection, or introgression, with *F*_ST_ ≤5%, *d*_XY_ ≤5%, and *π* ≤5%, where the reduction in all measures may reflect conserved regions under purifying selection, shared sweeps, or gene flow.

To identify haplotypes that are longer than expected, |iHS| was calculated in 10‐kb non‐overlapping windows utilizing the *rehh* package v3.2.2 in R (Gautier et al., 2017; Gautier & Vitalis, 2012). The phased VCF files, along with the inferred ancestral allelic states described above, were used for conducting a haplotype genome scan. The integrated Extended Haplotype Homozygosity (iHH) values were computed using the *scan_hh* function, followed by the calculation of iHS through the *ihh2ihs* function. Finally, the iHS values of the non‐overlapping 10‐kb windows with at least two markers were calculated using *calc_candidate_regions* function, and the absolute values of iHS were used for subsequent analyses. For window size in each scenario, a null distribution was generated from 1000 randomizations of *d*_XY_, and empirical *P*‐values were calculated as the proportion of randomized statistics at least as extreme as the observed value (two‐sided permutation test). For statistical comparisons of *ρ*, Tajima's *D*, and |iHS|, 3600 randomly selected windows were used as background windows and to compare with the observed values in two‐tailed Welch's *t*‐test.

## CONFLICT OF INTEREST

The authors declare that they have no conflicts of interest.

## DECLARATION OF AI USE

The authors used ChatGPT to assist with language editing, clarity improvement, and reorganization of the manuscript text. The AI tool was used iteratively to refine phrasing and structure, while the scientific content, including all ideas, data interpretation, and conclusions, originated from the authors. All AI‐assisted text was carefully reviewed, edited, and validated by the authors to ensure accuracy, originality, and consistency with the underlying research. The authors take full responsibility for the final content of the manuscript.

## Supporting information


**Table S1.** Metadata for the 100 individuals of three *Pinus* species sequenced in this study.
**Table S2.** Site number of different site categories in dataset in this study and reference genome (*Pinus tabuliformis* V1.0).
**Table S3.** Mean nucleotide diversity (*π*) and site number of different site categories in *Pinus nigra* from different data and methods.
**Table S4.** Inferred demographic parameters of the best‐fitting demographic model for *Pinus banksiana* and *P. contorta*.

**Figure S1.** Sampling localities (details in Table S1) and distribution areas of *Pinus banksiana*, *P. contorta*, and *P. nigra*.
**Figure S2.** The private and shared SNPs of *Pinus banksiana*, *P. contorta*, and *P. nigra*.
**Figure S3.** Genome‐wide patterns of nucleotide diversity (*π*) displayed across 10‐kb non‐overlapping windows for the three *Pinus* species.
**Figure S4.** Genome‐wide patterns of nucleotide diversity (*π*) displayed across 100‐kb non‐overlapping windows for the three *Pinus* species.
**Figure S5.** Magnified view of the genome‐wide PCA plot, highlighting the distribution of *Pinus contorta* (A) and *P. nigra* (B), as shown in Figure 1A.
**Figure S6.** Decay of linkage disequilibrium (LD) of the three *Pinus* species.
**Figure S7.** Genome‐wide patterns of population‐scaled recombination rate (*ρ*) over 10‐Mb non‐overlapping windows among three *Pinus* species.
**Figure S8.** Genome‐wide patterns of population‐scaled recombination rate (*ρ*) over 1‐Mb non‐overlapping windows among three *Pinus* species.
**Figure S9.** Correlations of population‐scaled recombination rate (*ρ*) over 10‐ and 1‐Mb non‐overlapping windows between pairwise comparisons of the three *Pinus* species.
**Figure S10.** Correlations between population‐scaled recombination rate (*ρ*) and nucleotide diversity (*π*) among three *Pinus* species over 10‐ and 1‐Mb non‐overlapping windows.
**Figure S11.** Correlations between population‐scaled recombination rate (*ρ*) and gene number among three *Pinus* species over 10‐ and 1‐Mb non‐overlapping windows.
**Figure S12.** Correlations between population‐scaled recombination rate (*ρ*) and accumulated gene length in 10‐ and 1‐Mb non‐overlapping windows among three *Pinus* species.
**Figure S13.** Correlations between population‐scaled recombination rate (*ρ*) and accumulated exon length in 10‐ and 1‐Mb non‐overlapping windows among three *Pinus* species.
**Figure S14.** Correlations between population‐scaled recombination rate (*ρ*) and accumulated intron length in 10‐ and 1‐Mb non‐overlapping windows among three *Pinus* species.
**Figure S15.** Correlations between population‐scaled recombination rate (*ρ*) and transposable element (TE) density among three *Pinus* species.
**Figure S16.** The distributions of estimates of *d*_XY_ and *F*_ST_ between the three *Pinus* species.
**Figure S17.** Genome‐wide correlation analysis for *d*_XY_ and *F*_ST_ between the three *Pinus* species pairs.

## Data Availability

The genome sequencing data generated in this study have been deposited in the Sequence Read Archive (SRA) at the National Center for Biotechnology Information (NCBI) under the BioProject accession number PRJNA945376 (https://www.ncbi.nlm.nih.gov/bioproject/?term=PRJNA945376).
