# Supplementary material for: Natural selection and recombination interact to structure genome‐wide variation in pines
Source: Plant J. 2026 Apr 14;126(1):e70866. doi: 10.1111/tpj.70866 (PMC13078855; doi:10.1111/tpj.70866)
Supplement: Supplementary file 2 — Figure S1. Sampling localities (details in Table S1) and distribution areas of Pinus banksiana, P. contorta, and P. nigra. Figure S2. The private and shared SNPs of Pinus banksiana, P. contorta, and P. nigra. Figure S3. Genome‐wide patterns of nucleotide diversity (π) displayed across 10‐kb non‐overlapping windows for the three Pinus species. Figure S4. Genome‐wide patterns of nucleotide diversity (π) displayed across 100‐kb non‐overlapping windows for the three Pinus species. Figure S5. Magnified view of the genome‐wide PCA plot, highlighting the distribution of Pinus contorta (A) and P. nigra (B), as shown in Figure 1A. Figure S6. Decay of linkage disequilibrium (LD) of the three Pinus species. Figure S7. Genome‐wide patterns of population‐scaled recombination rate (ρ) over 10‐Mb non‐overlapping windows among three Pinus species. Figure S8. Genome‐wide patterns of population‐scaled recombination rate (ρ) over 1‐Mb non‐overlapping windows among three Pinus species. Figure S9. Correlations of population‐scaled recombination rate (ρ) over 10‐ and 1‐Mb non‐overlapping windows between pairwise comparisons of the three Pinus species. Figure S10. Correlations between population‐scaled recombination rate (ρ) and nucleotide diversity (π) among three Pinus species over 10‐ and 1‐Mb non‐overlapping windows. Figure S11. Correlations between population‐scaled recombination rate (ρ) and gene number among three Pinus species over 10‐ and 1‐Mb non‐overlapping windows. Figure S12. Correlations between population‐scaled recombination rate (ρ) and accumulated gene length in 10‐ and 1‐Mb non‐overlapping windows among three Pinus species. Figure S13. Correlations between population‐scaled recombination rate (ρ) and accumulated exon length in 10‐ and 1‐Mb non‐overlapping windows among three Pinus species. Figure S14. Correlations between population‐scaled recombination rate (ρ) and accumulated intron length in 10‐ and 1‐Mb non‐overlapping windows among three Pinus species. Figure S15. [file TPJ-126-e70866-s001.docx]

**Supplementary Figures**

**Table of Contents**

Fig. S1. Sampling localities (details in Table S1) and distribution areas of *Pinus banksiana*, *P. contorta* and *P. nigra*.

Fig. S2. The private and shared SNPs of *Pinus banksiana*, *P. contorta*, and *P. nigra*.

Fig. S3. Genome-wide patterns of nucleotide diversity (*π*) displayed across 10-kb non-overlapping windows for the three *Pinus* species.

Fig. S4. Genome-wide patterns of nucleotide diversity (*π*) displayed across 100-kb non-overlapping windows for the three *Pinus* species.

Fig. S5. Magnified view of the genome-wide PCA plot, highlighting the distribution of *Pinus contorta* (A) and *P. nigra* (B), as shown in Fig. 1A.

Fig. S6. Decay of linkage disequilibrium (LD) of the three *Pinus* species.

Fig. S7. Genome-wide patterns of population-scaled recombination rate (*ρ*) over 10-Mb non-overlapping windows among three *Pinus* species.

Fig. S8. Genome-wide patterns of population-scaled recombination rate (*ρ*) over 1-Mb non-overlapping windows among three *Pinus* species.

Fig. S9. Correlations of population-scaled recombination rate (*ρ*) over 10-Mb and 1-Mb non-overlapping windows between pairwise comparisons of the three *Pinus* species.

Fig. S10. Correlations between population-scaled recombination rate (*ρ*) and nucleotide diversity (*π*) among three *Pinus* species over 10-Mb and 1-Mb non-overlapping windows.

Fig. S11. Correlations between population-scaled recombination rate (*ρ*) and gene number among three *Pinus* species over 10-Mb and 1-Mb non-overlapping windows.

Fig. S12. Correlations between population-scaled recombination rate (*ρ*) and accumulated gene length in 10-Mb and 1-Mb non-overlapping windows among three *Pinus* species.

Fig. S13. Correlations between population-scaled recombination rate (*ρ*) and accumulated exon length in 10-Mb and 1-Mb non-overlapping windows among three *Pinus* species.

Fig. S14. Correlations between population-scaled recombination rate (*ρ*) and accumulated intron length in 10-Mb and 1-Mb non-overlapping windows among three *Pinus* species.

Fig. S15. Correlations between population-scaled recombination rate (*ρ*) and transposable element (TE) length (upper panels) and TE density (lower panels) among three *Pinus* species.

Fig. S16. The distributions of estimates of *d*_XY_ and *F*_ST_ between the three *Pinus* species.

Fig. S17. Genome-wide correlation analysis for *d*_XY_ and *F*_ST_ between the three *Pinus* species pairs.


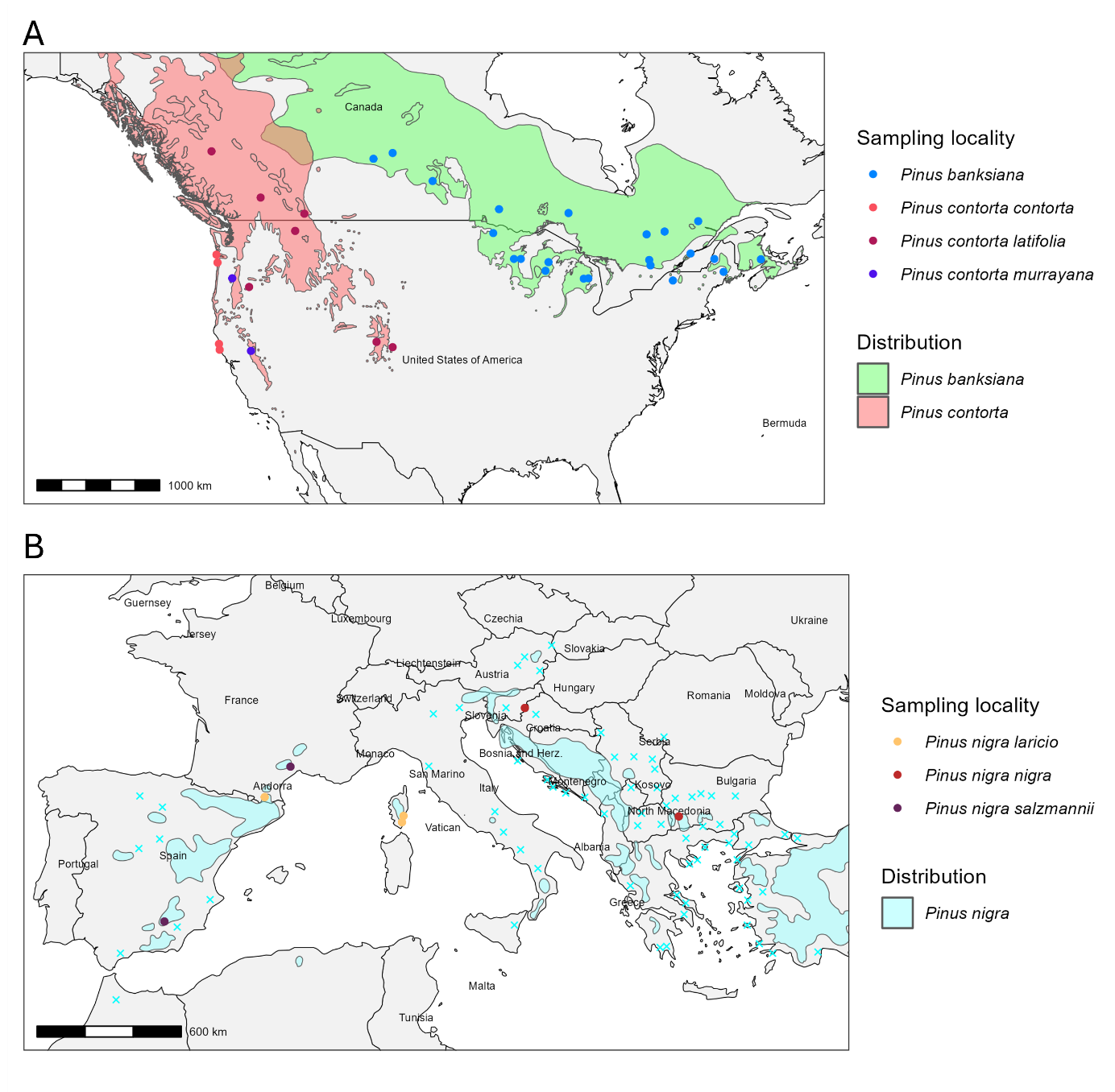


Fig. S1. Sampling localities (details in Table S1) and distribution areas of *Pinus banksiana* (green), *P. contorta* (red) and *P. nigra* (blue).


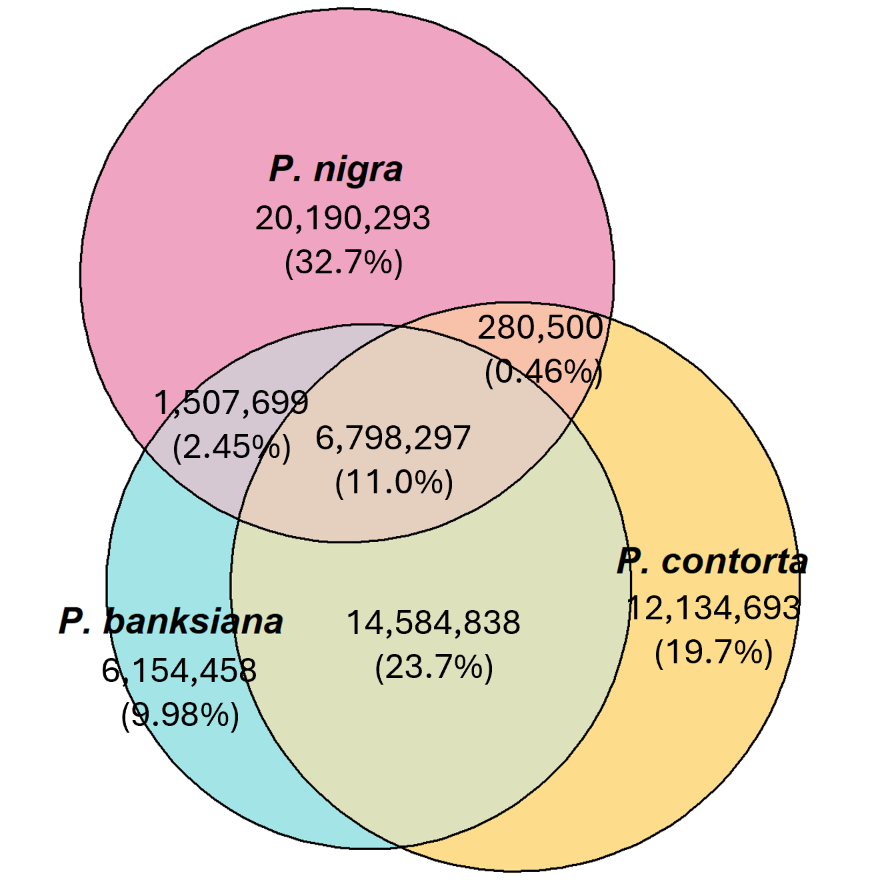


Fig. S2. The private and shared SNPs of *Pinus banksiana*, *P. contorta*, and *P. nigra*.


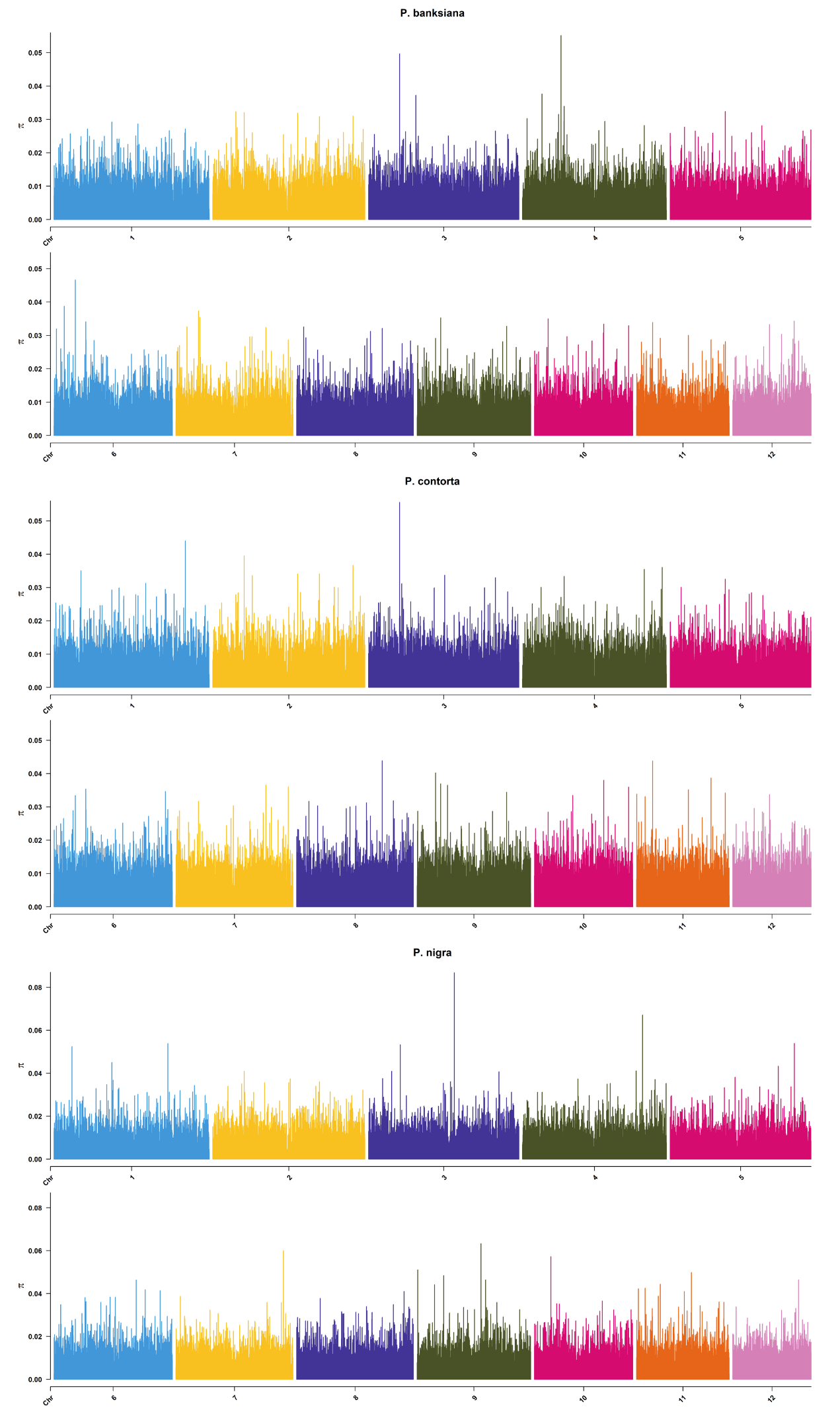


Fig. S3. Genome-wide patterns of nucleotide diversity (*π*) displayed across 10-kb non-overlapping windows for the three *Pinus* species.


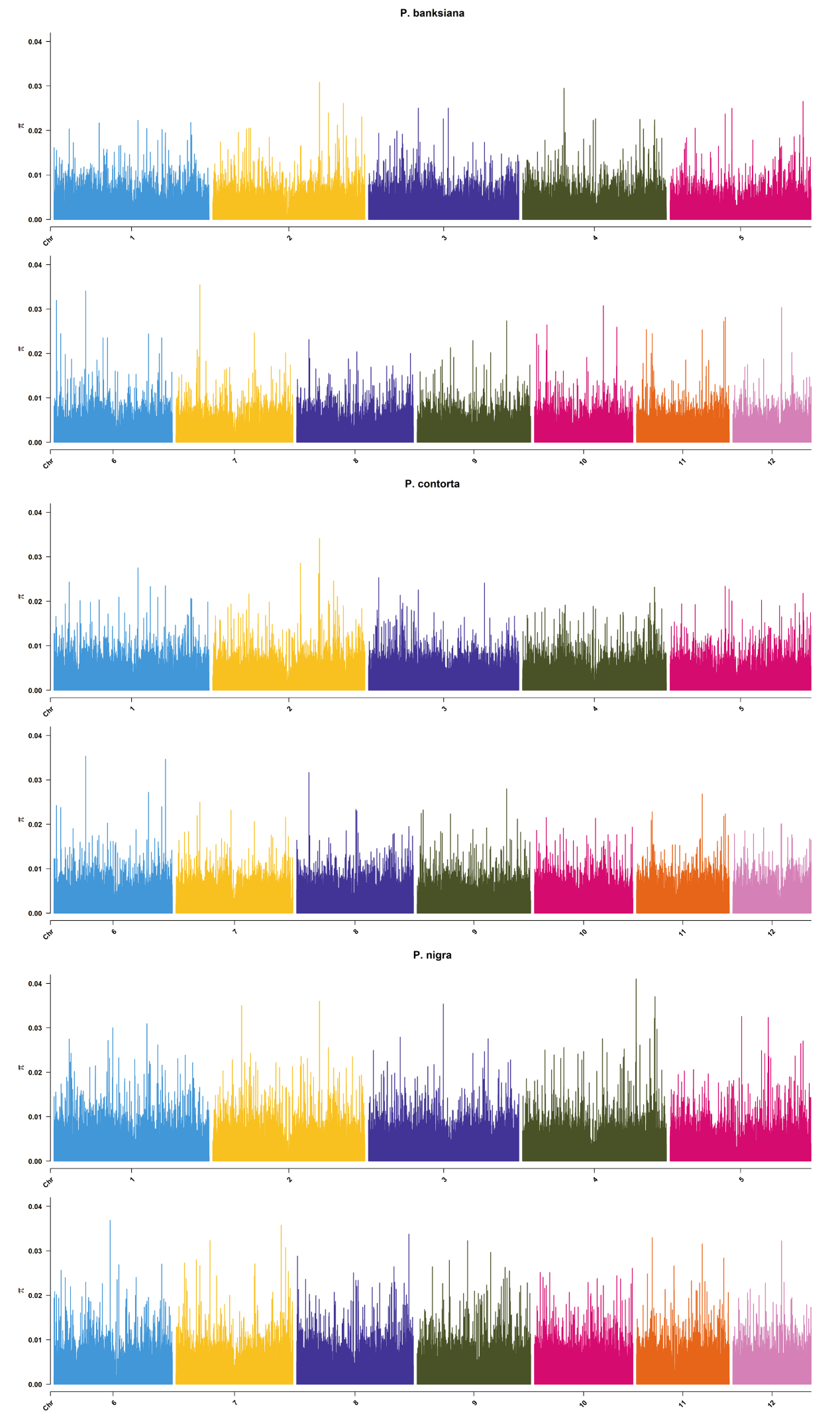


Fig. S4. Genome-wide patterns of nucleotide diversity (*π*) displayed across 100-kb non-overlapping windows for the three *Pinus* species.


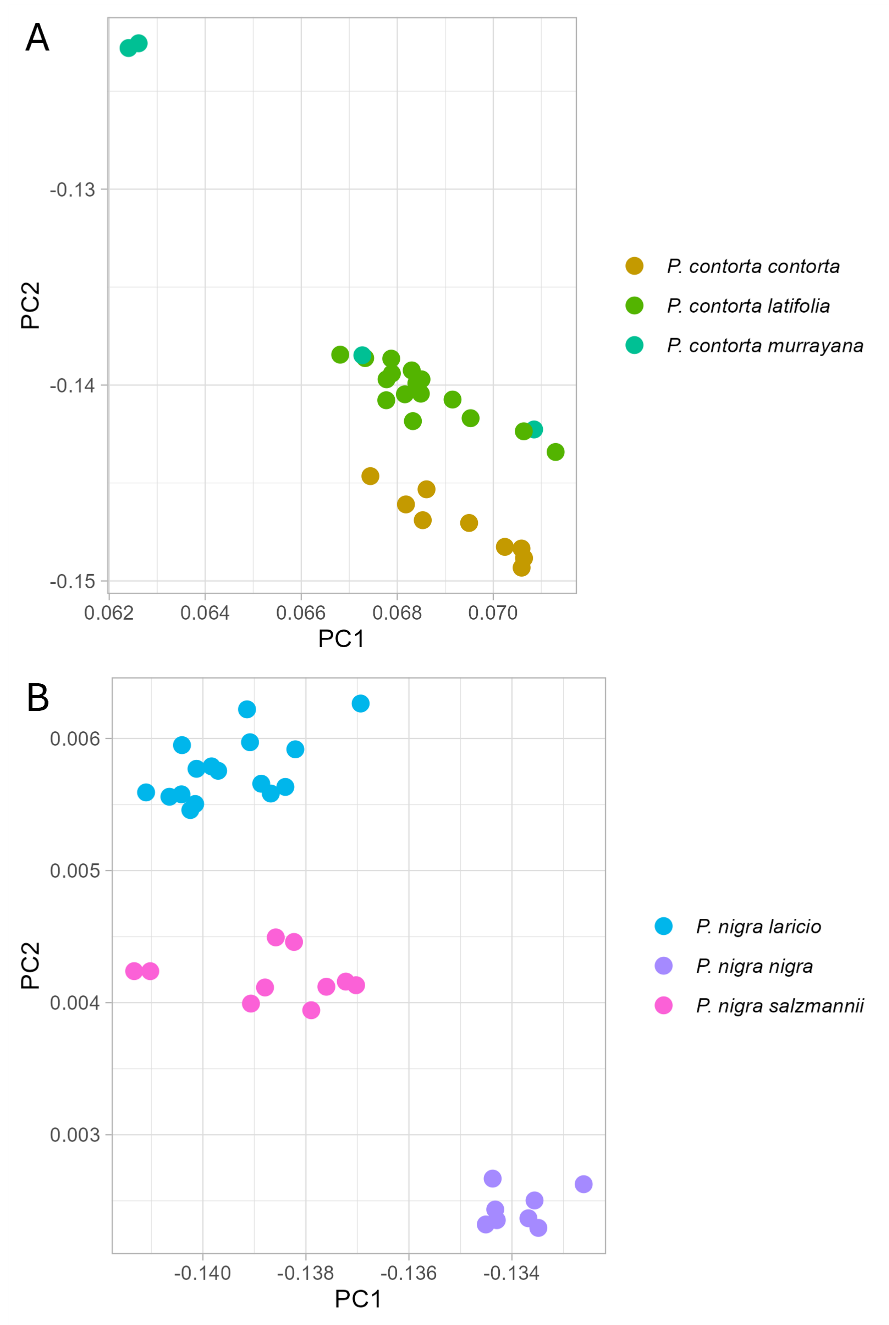


Fig. S5. Magnified view of the genome-wide PCA plot, highlighting the distribution of *Pinus contorta* (A) and *P. nigra* (B), as shown in Fig. 1A.


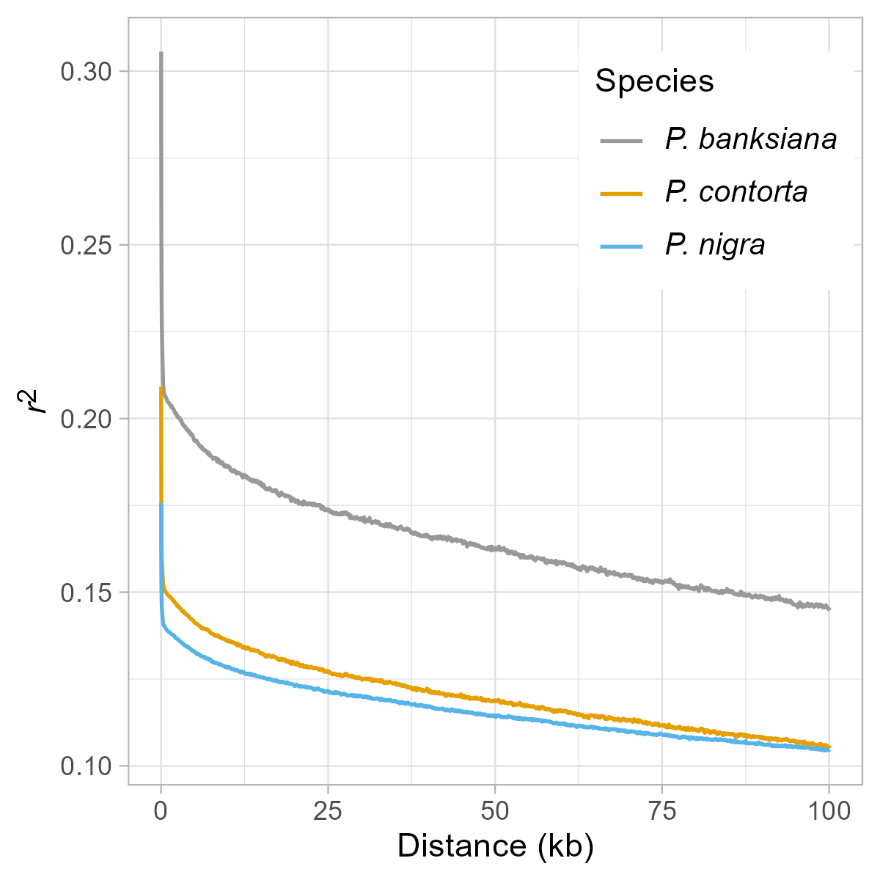


Fig. S6. Decay of linkage disequilibrium (LD) of the three *Pinus* species.


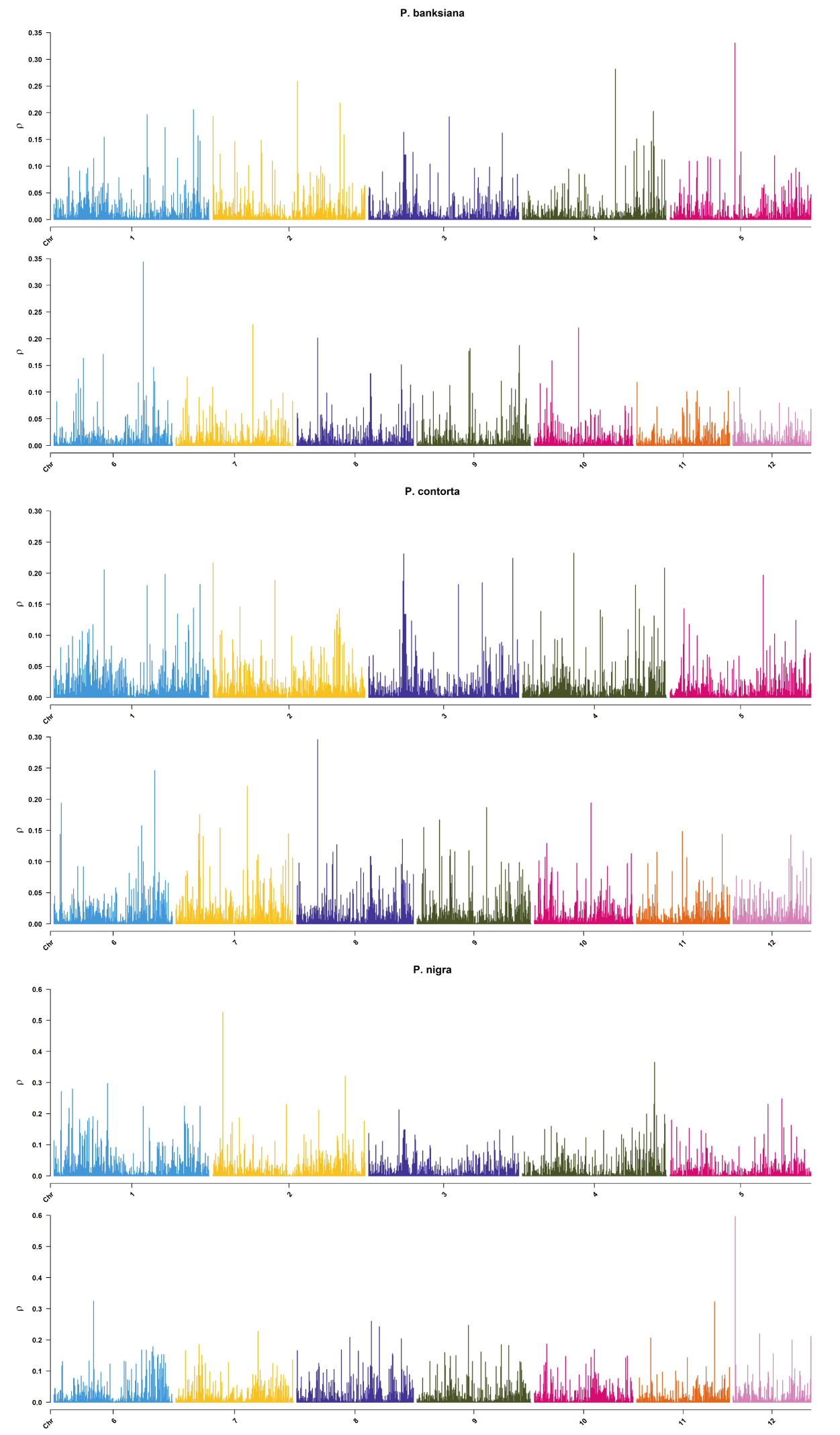


Fig. S7. Genome-wide patterns of population-scaled recombination rate (*ρ*) over 10-Mb non-overlapping windows among three *Pinus* species. (A) *P. banksiana*, (B) *P. contorta*, and (C) *P. nigra*.


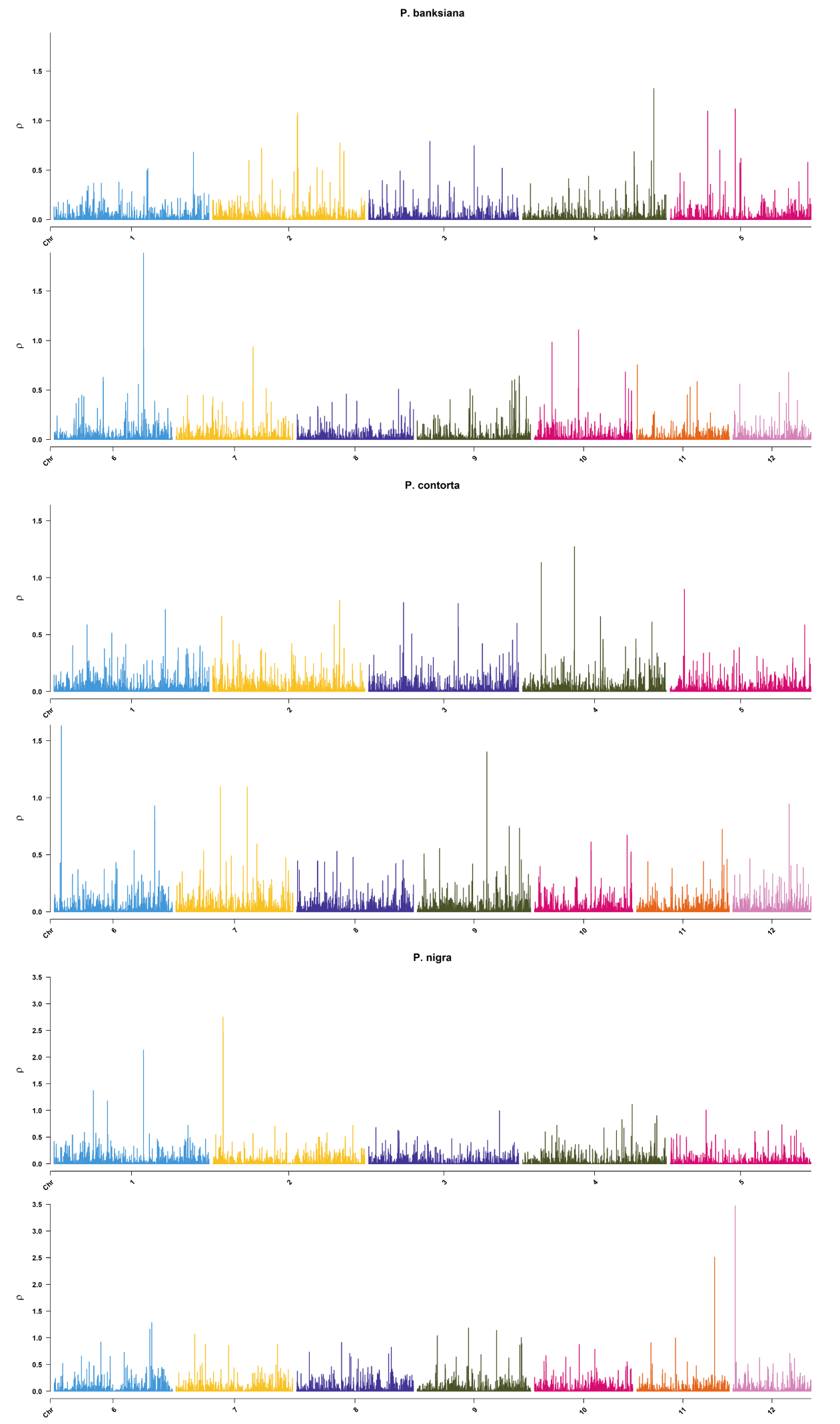


Fig. S8. Genome-wide patterns of population-scaled recombination rate (*ρ*) over 1-Mb non-overlapping windows among three *Pinus* species. (A) *P. banksiana*, (B) *P. contorta*, and (C) *P. nigra*.


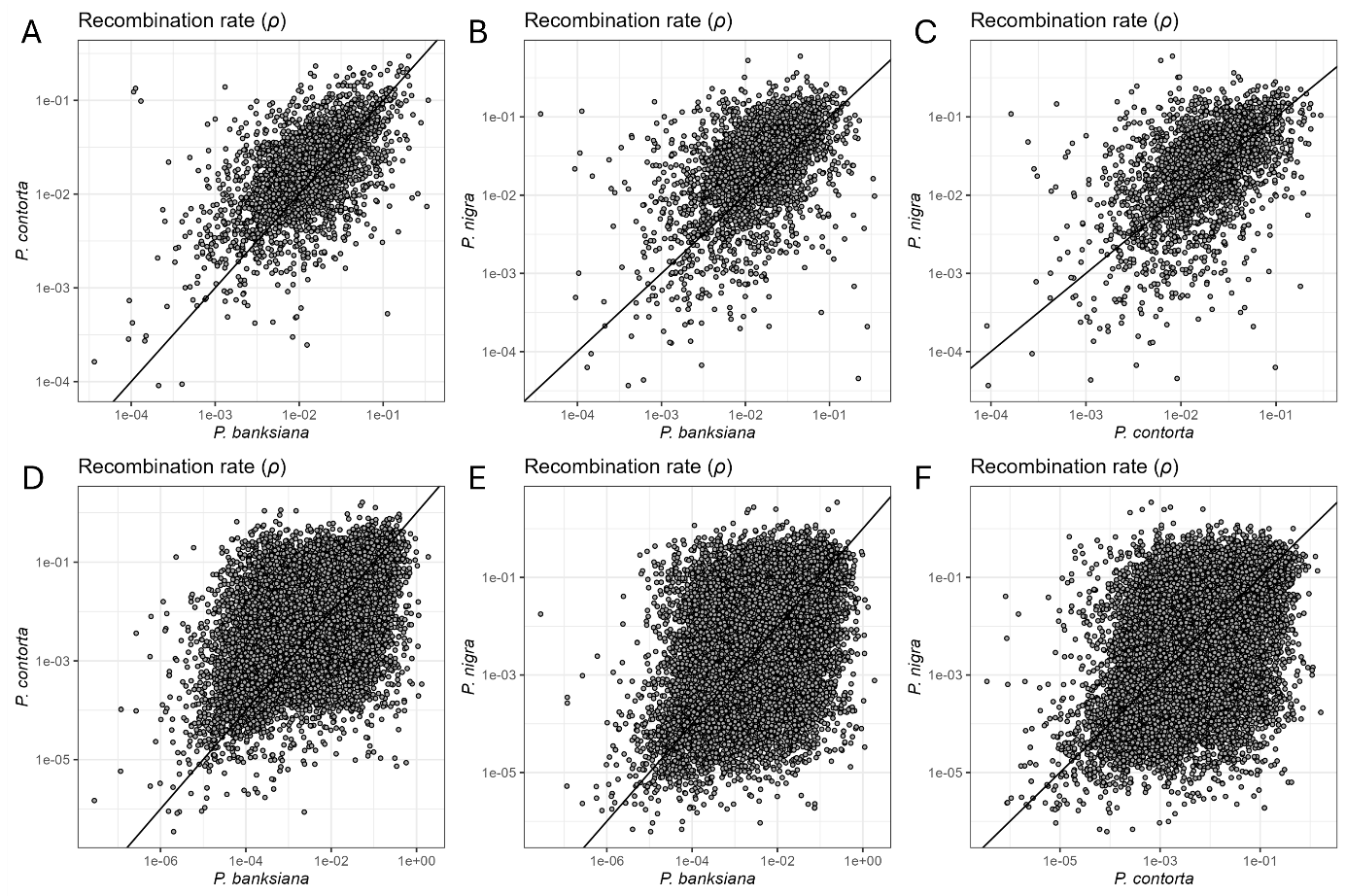


Fig. S9. Correlations of population-scaled recombination rate (*ρ*) over 10-Mb and 1-Mb non-overlapping windows between pairwise comparisons of the three *Pinus* species. Each dot represents a 10-Mb non-overlapping window (A–C), or a 1-Mb non-overlapping window (D–F). The black line in each plot represents a regression line with a zero intercept and a slope of one. All pairwise comparisons show a *P*-value < 2.2e-16.


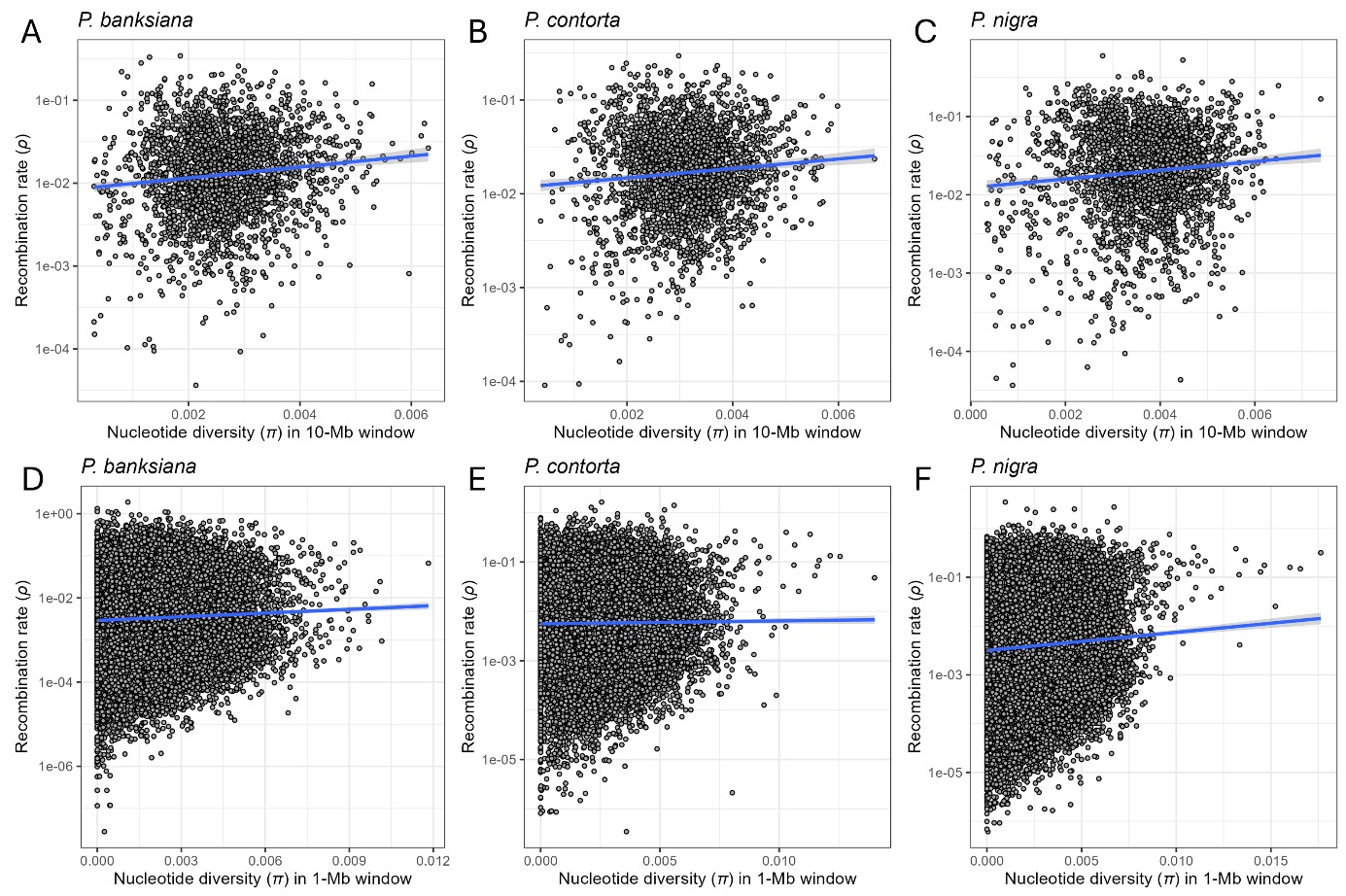


Fig. S10. Correlations between population-scaled recombination rate (*ρ*) and nucleotide diversity (*π*) among three *Pinus* species over 10-Mb and 1-Mb non-overlapping windows. Each dot represents a 10-Mb non-overlapping window (A–C), or a 1-Mb non-overlapping window (D–F). (A–D, F) *P*-value < 4.7e-06; (E) *P*-value = 0.115.


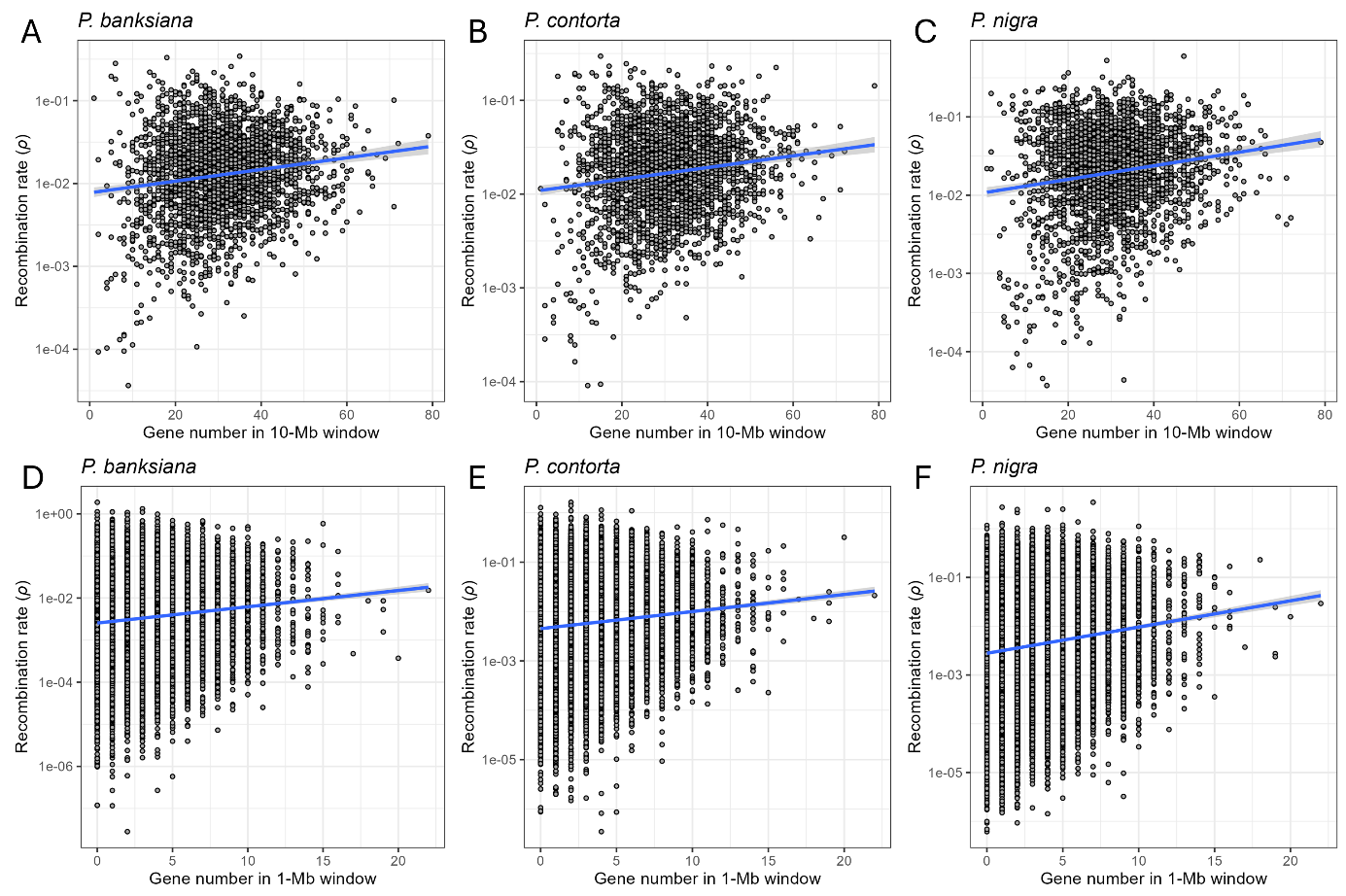


Fig. S11. Correlations between population-scaled recombination rate (*ρ*) and gene number among three *Pinus* species over 10-Mb and 1-Mb non-overlapping windows. Each dot represents a 10-Mb non-overlapping window (A–C), or a 1-Mb non-overlapping window (D–F). (A–C) *P*-value < 3.8e-13; (D–F) *P*-value < 2.2e-16.


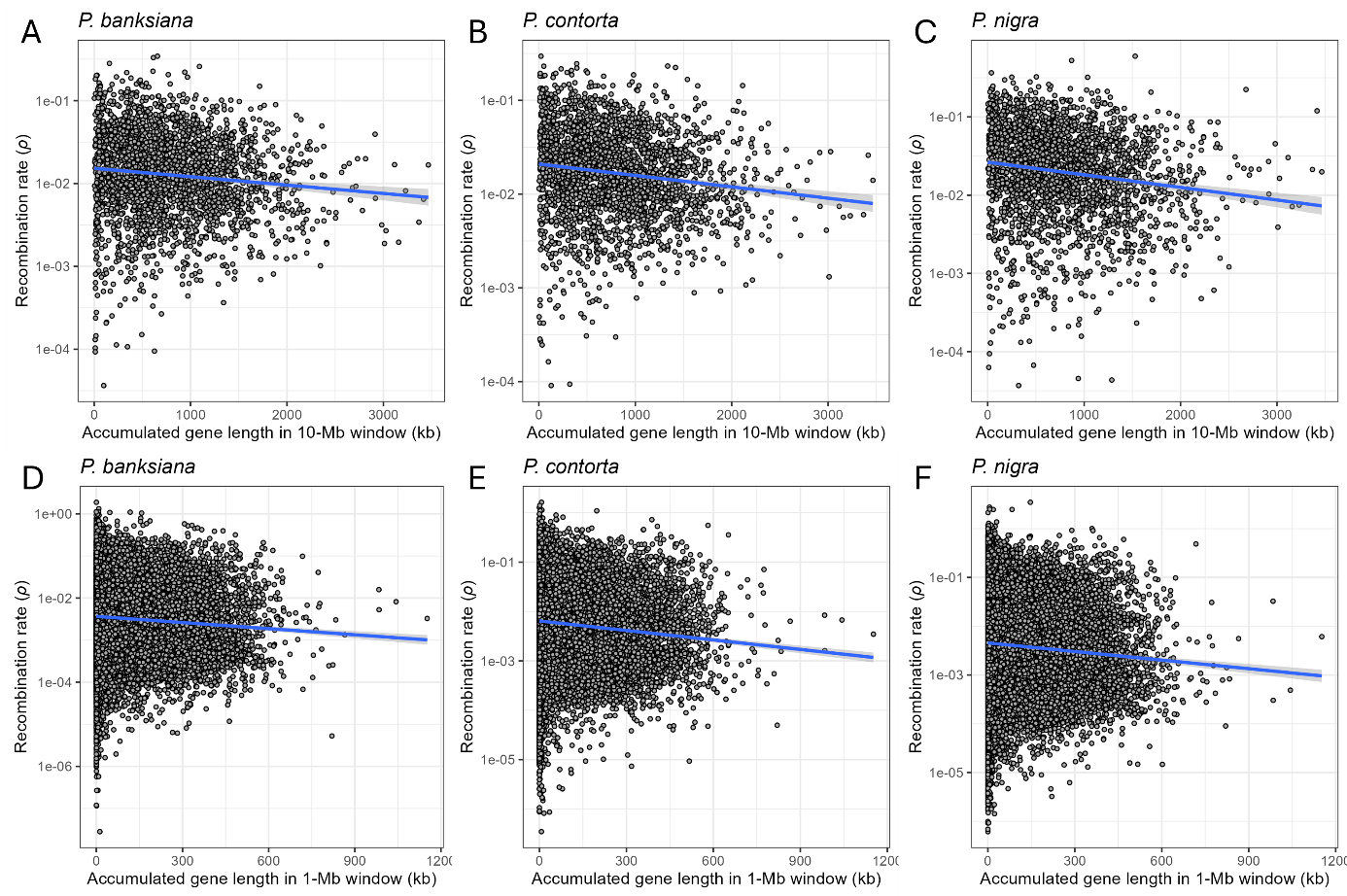


Fig. S12. Correlations between population-scaled recombination rate (*ρ*) and accumulated gene length in 10-Mb and 1-Mb non-overlapping windows among three *Pinus* species. Each dot represents a 10-Mb non-overlapping window (A–C), or a 1-Mb non-overlapping window (D–F). (A–C) *P*-value < 5.9e-08; (D–F) *P*-value < 2.2e-16.


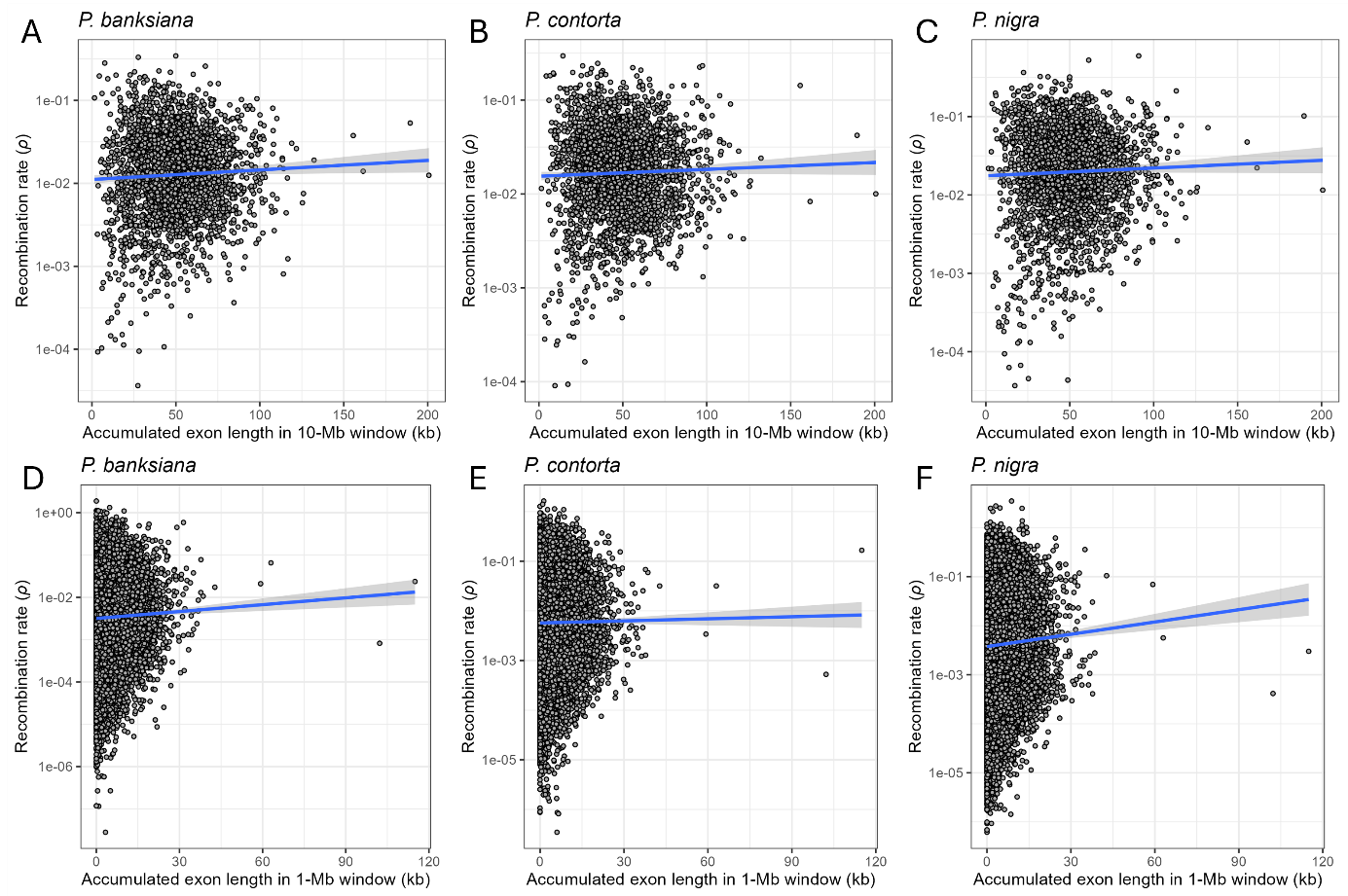


Fig. S13. Correlations between population-scaled recombination rate (*ρ*) and accumulated exon length in 10-Mb and 1-Mb non-overlapping windows among three *Pinus* species. Each dot represents a 10-Mb non-overlapping window (A–C), or a 1-Mb non-overlapping window (D–F). (A) *P*-value = 0.019; (B) *P*-value = 0.100; (C) *P*-value = 0.076; (D) *P*-value = 7.6e-05; (E) *P*-value = 0.251; (F) *P*-value = 5.1e-08.


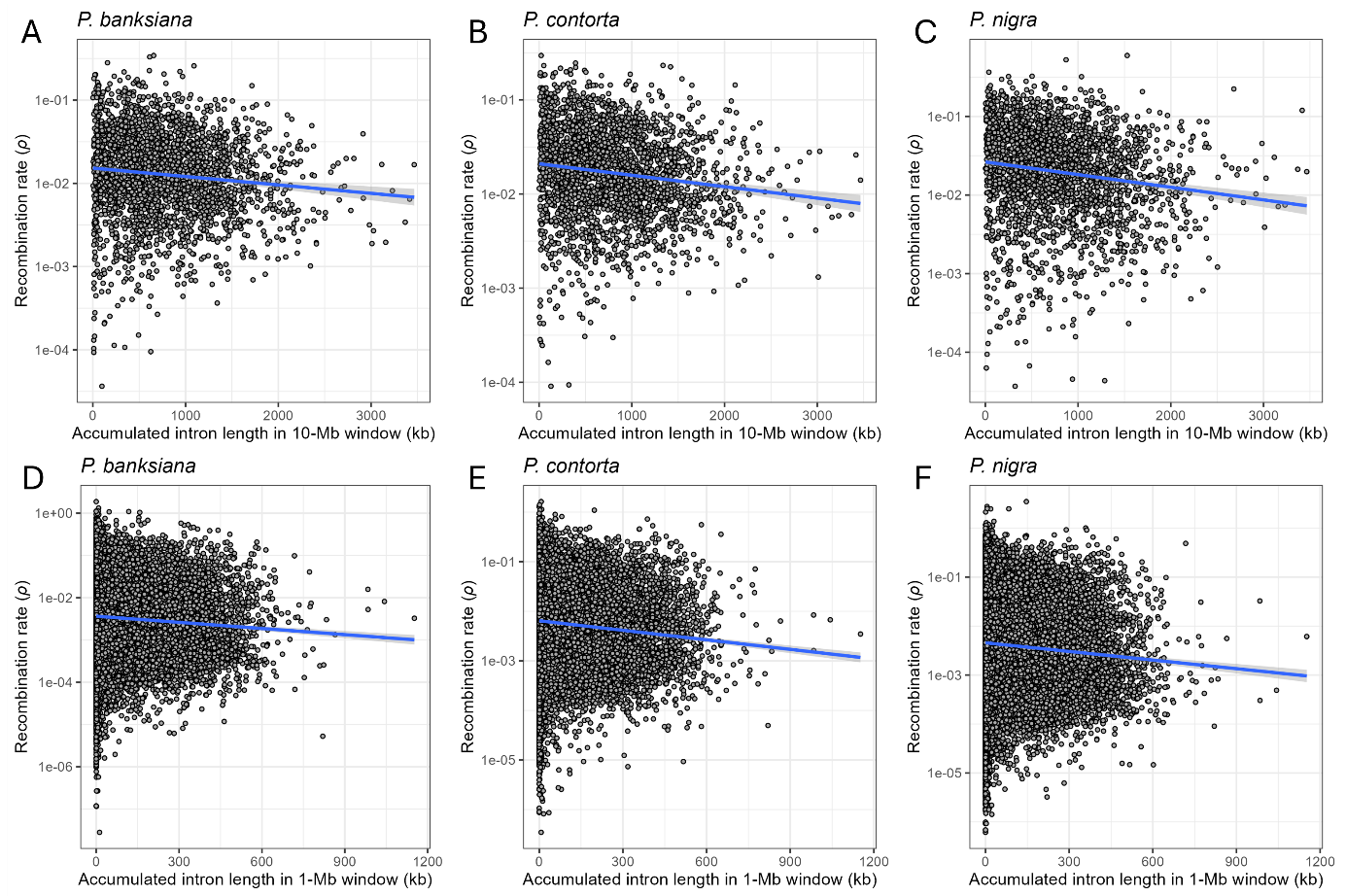


Fig. S14. Correlations between population-scaled recombination rate (*ρ*) and accumulated intron length in 10-Mb and 1-Mb non-overlapping windows among three *Pinus* species. Each dot represents a 10-Mb non-overlapping window (A–C), or a 1-Mb non-overlapping window (D–F). (A–C) *P*-value < 2.0e-08; (D–F) *P*-value < 2.2e-16.


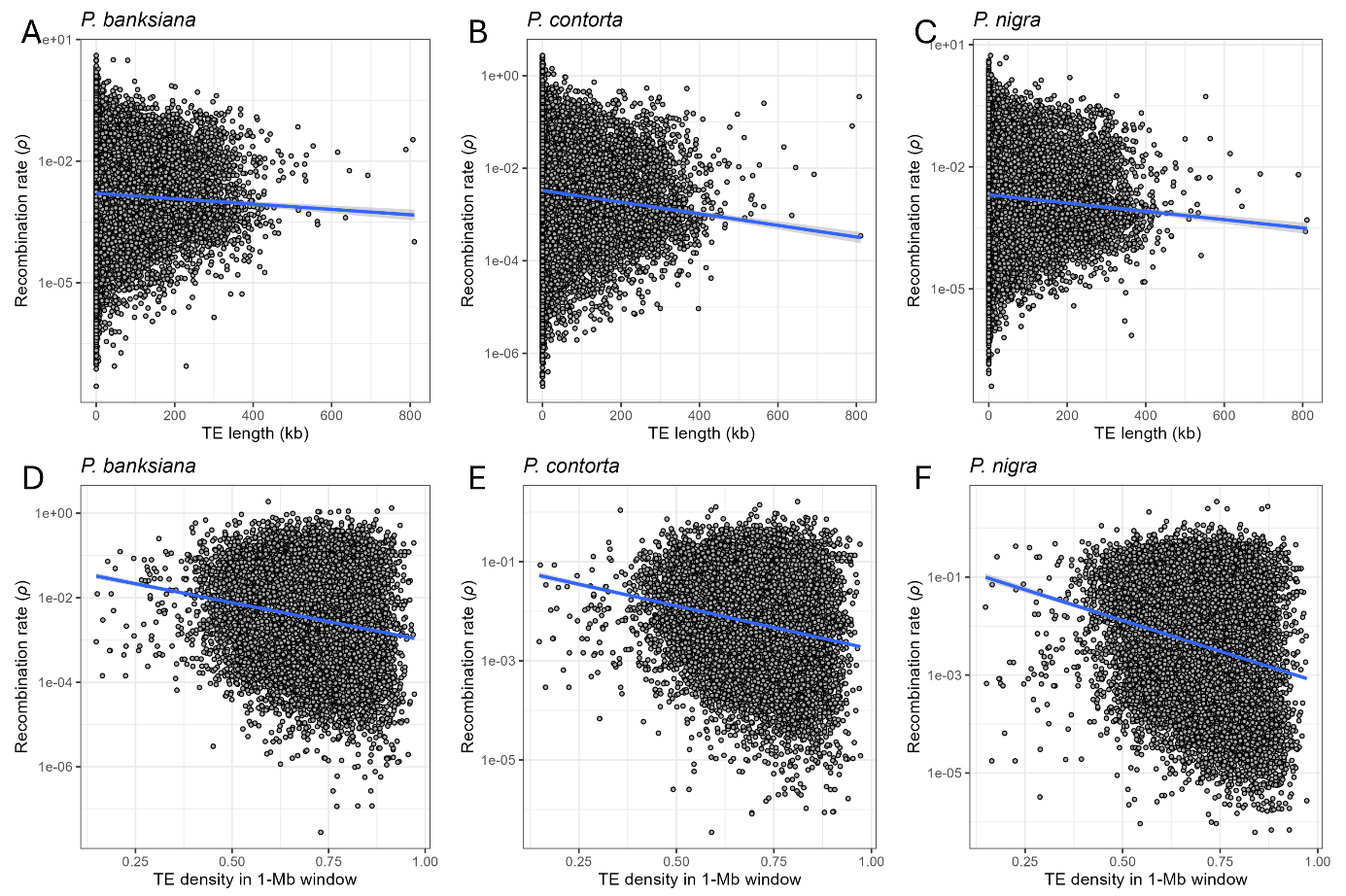


Fig. S15. Correlations between population-scaled recombination rate (*ρ*) and transposable element (TE) length (upper panels) and TE density (lower panels) among three *Pinus* species. (A–C) Each dot represents an annotated gene of the reference genome. *P*-value < 9.5e-12; (D–F) Each dot represents a 1-Mb non-overlapping window. *P*-value < 2.2e-16.


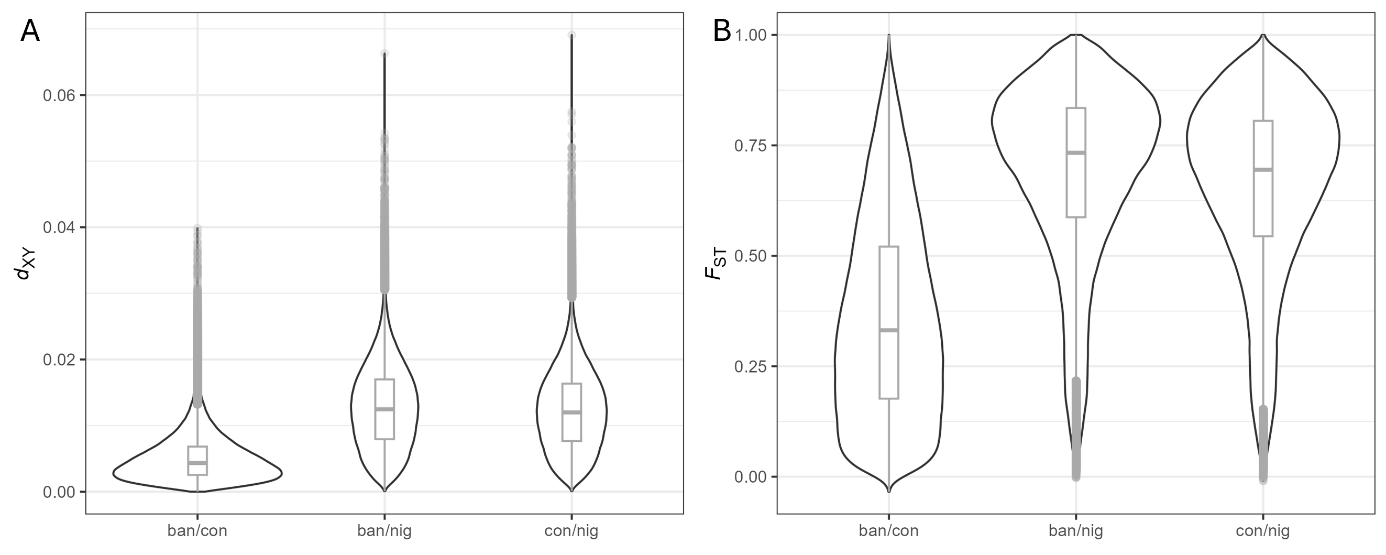


Fig. S16. The distributions of estimates of *d*_XY_ and *F*_ST_ between the three *Pinus* species. (A) absolute nucleotide divergence, *d*_XY_; (B) relative genetic divergence, *F*_ST_. ban, *P. banksiana*; con, *P. contorta*; nig, *P. nigra*.


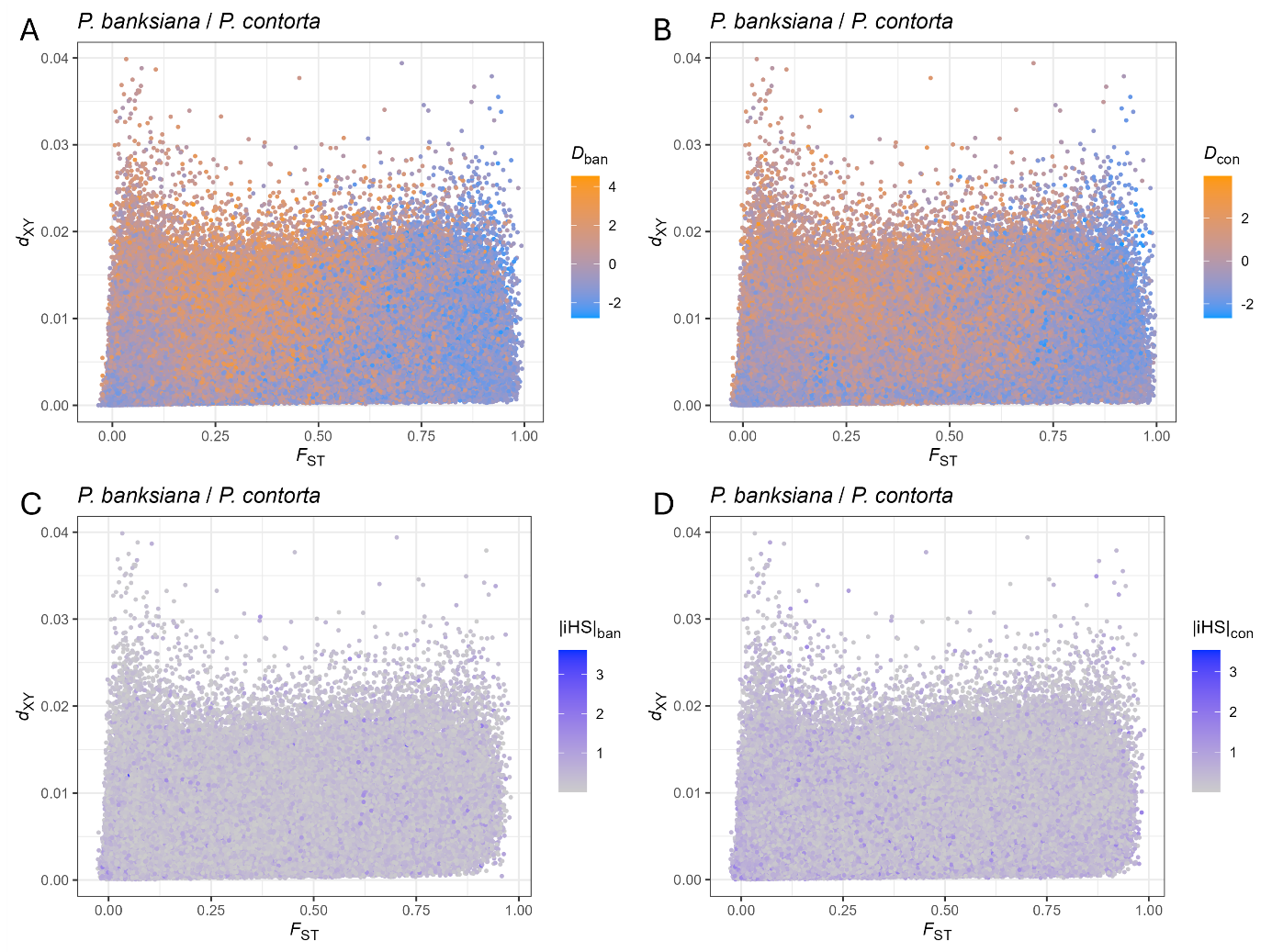


Fig. S17. Genome-wide correlation analysis for *d*_XY_ and *F*_ST_ between the three *Pinus* species pairs. Each dot represents a 10-kb non-overlapping window. (A) and (B) with Tajima’s *D* value in color; (C) and (D) with |iHS| value in color.
